# Genome-Wide PhoB Binding and Gene Expression Profiles Reveal the Hierarchical Gene Regulatory Network of Phosphate Starvation in *Escherichia coli*

**DOI:** 10.1371/journal.pone.0047314

**Published:** 2012-10-05

**Authors:** Chi Yang, Tzu-Wen Huang, Shiau-Yi Wen, Chun-Yang Chang, Shih-Feng Tsai, Whei-Fen Wu, Chuan-Hsiung Chang

**Affiliations:** 1 Institute of Biomedical Informatics, Center for Systems and Synthetic Biology, National Yang Ming University, Taipei, Taiwan; 2 Center for Systems and Synthetic Biology, National Yang Ming University, Taipei, Taiwan; 3 Division of Molecular and Genomic Medicine, National Health Research Institutes, Miaoli, Taiwan; 4 Department of Agricultural Chemistry, National Taiwan University, Taipei, Taiwan; CRS4, Italy

## Abstract

The phosphate starvation response in bacteria has been studied extensively for the past few decades and the phosphate-limiting signal is known to be mediated via the PhoBR two-component system. However, the global DNA binding profile of the response regulator PhoB and the PhoB downstream responses are currently unclear. In this study, chromatin immunoprecipitation for PhoB was combined with high-density tiling array (ChIP-chip) as well as gene expression microarray to reveal the first global down-stream responses of the responding regulator, PhoB in *E. coli*. Based on our ChIP-chip experimental data, forty-three binding sites were identified throughout the genome and the known PhoB binding pattern was updated by identifying the conserved pattern from these sites. From the gene expression microarray data analysis, 287 differentially expressed genes were identified in the presence of PhoB activity. By comparing the results obtained from our ChIP-chip and microarray experiments, we were also able to identify genes that were directly or indirectly affected through PhoB regulation. Nineteen out of these 287 differentially expressed genes were identified as the genes directly regulated by PhoB. Seven of the 19 directly regulated genes (including *phoB*) are transcriptional regulators. These transcriptional regulators then further pass the signal of phosphate starvation down to the remaining differentially expressed genes. Our results unveiled the genome-wide binding profile of PhoB and the downstream responses under phosphate starvation. We also present the hierarchical structure of the phosphate sensing regulatory network. The data suggest that PhoB plays protective roles in membrane integrity and oxidative stress reduction during phosphate starvation.

## Introduction

Phosphate participates in many important cellular processes [1] such as energy metabolism, and the construction of genetic molecules and organelles including cell membranes. Since the concentration of phosphate is usually low in natural environments, many bacteria have evolved to sense this essential nutrient and to adapt to phosphate-limiting conditions. Several transcriptomics and proteomics studies had been done to reveal bacteria adaptation in a diverse range of bacteria including *Bacillus subtilis* [2], *Corynebacterium glutamicum* [3], *Escherichia coli* [4], [5], *Prochlorococcus marinus* [6], *Sinorhizobium meliloti* [7] *and Vibrio cholera* [8].

In *E. coli*, phosphate sensing had been reported to be performed by a seven-component apparatus [9]. The sensor kinase of this machinery, PhoR, plays an important role to pass the limited environmental phosphate signal to its response regulator, PhoB. During phosphate starvation, PhoR dimer is autophosphorylated on one histidine residue of each monomer. This phospho-PhoR dimer has the kinase activity that can transfer the two phosphoryl groups to the aspartate residue in each of the PhoB monomers [1]. The phospho-PhoB dimer is the active form of the transcriptional factor that recognizes the previously characterized PhoB recognition consensus sequence CTGTCAT-A(AT)A(TA)-CTGT(CA)A(CT) (Pho box) and regulates its target genes [1], [10], [11]. In response to phosphate limitation, PhoB binds to the Pho box and transmits the phosphate-limiting signal to downstream responding genes.

To date, thirty-one responding genes composed of nine transcription units are known to be regulated by PhoB, while several other genes lack direct evidence of PhoB binding in *E. coli* [9]. However, previously reported proteomics data of *E. coli* indicate that the expression of around 400 proteins varied in a comparison between excess and limited phosphate conditions [5]. Thus, studying the genome-wide regulation exercised by PhoB in response to phosphate starvation is required to understand the underlying mechanisms of bacterial adaptation to phosphate starvation.

In this study, we combined ChIP-chip and gene expression microarray experiments, for the first time, to present the global responses of *E. coli* to phosphate starvation through the PhoR/PhoB two-component system. This integrative genome-wide approach allowed us to identify 54 PhoB binding targets and 287 differentially expressed genes in the presence of PhoB activity during phosphate starvation. These results indicate that PhoB directly regulates a group of genes which contain distinct transcriptional regulators and further indirectly influences other genes. A specific group of genes involved in the functions of transportation and metabolism for membrane protection have also been identified.

## Results and Discussion

### Genome-wide mapping of PhoB binding profiles

**Figure 1:**
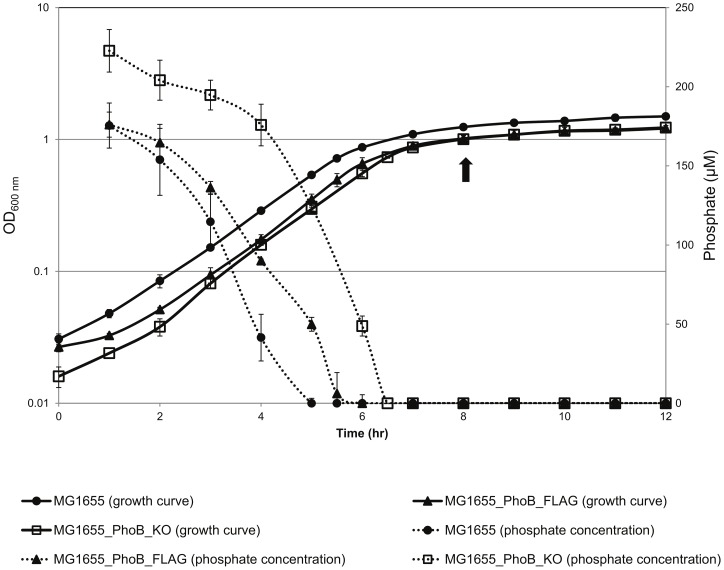
Cultivation of *E. coli* MG1655, MG1655_PhoB_FLAG, and MG1655_PhoB_KO strains under the phosphate limiting condition. The dashed lines indicate the phosphate consumption of MG1655 (filled circles), MG1655_PhoB_FLAG (filled triangles) and MG1655_PhoB_KO (hollow rectangles). The solid lines show the growth curves of the three strains. Bacterial cultures were harvested at an OD_600_ value of 1.0 (indicated by the black arrow) for both the ChIP-chip and gene expression microarray experiments. For the reporter gene assay, transformed cells were also harvested at an OD_600_ value of 1.0.

We applied the ChIP-chip techniques to measure the binding of PhoB across the whole genome under the phosphate-limiting condition (Figure 1). The PhoB-FLAG expressing strain (MG1655_PhoB_FLAG) and the wild type strain (MG1655), which contains no FLAG tag, were used as a comparison for the recognition of anti-FLAG antibody (Table 1). The activity of our PhoB-FLAG fusion protein in the MG1655_PhoB_FLAG strain was nearly the same as the activity of PhoB in the MG1655 wild type strain (see Figure S1). In this design, the genome-wide map of interactions between PhoB and *E. coli* genomic DNA was constructed (Figure S2A). Our ChIP-chip results contained six of the nine PhoB-regulating targets described in a recent review [9]. The six targets are *ugpB*, *phnC*, *phoA pstS*, *phoE* and *phoB* (Figure S2B). One possible reason that we were not able to detect all the previously described targets may be due to the differences in experimental conditions or the *in vivo*/*in vitro* experimental designs. Further investigation of significantly enriched regions revealed 43 significantly enriched peaks identified by a CMARRT package [12] with a controlled error rate set at 0.05 (see Methods for details). Other uncharacterized PhoB targets were also identified in our study, and the overall target genes were classified into six groups with functions involved in transcriptional regulation, transportation, metabolism, membrane structure, unknown function and pseudogene (Table 2).

**Table 1:** Bacterial strains used in this study.

Name	Description	References or resources
BL21		
BW25113	*lacI* ^q^, *rrnB*_T14_, Δ*lacZ*_WJ16_, *hsd*R514, Δ*araBAD*_AH33_, Δ*rhaBAD*_LD78_	[30]
MG1655	F-, lamda-, *ilvG*-, *rfb*-50, *rph*-1	[44]
MG1655_PhoB_FLAG	MG1655 encoding *phoB*-3xFLAG	Constructed in this study
MG1655_PhoB_KO	MG1655 *phoB*::Km^r^	Constructed in this study

Abbreviations: Kmr, kanamycin resistance.

**Table 2:** PhoB targets identified by analyzing ChIP-chip data.

Peak center	Gene^a^	Log2-ratio^b^	Sequence motif identified by MEME	Distance to the translation start site^c^
(A) Transcription regulation				
331900.5	*yahA*	0.7814	CATTAATATATCTGTGAC	291.5
347564.5	*prpR*	−1.9058	TTGCAACAATTATGAAAC	−52.5
**416096.5**	***phoB***	8.5176	**TTTTCATAAATCTGTCAT**	**−72.5**
594808.5	*cusR*	1.0307	AAATGACAAAATTGTCAT	−77.5
1445357.5	*feaR*	−1.4766	TTGCAACACAAATGCAAC	−43.5
1626323.5	*ydfH*	1.1505	TTGTCACAAAAAAGTGGT	−46.5
1975957.5	*flhD*	*-*	ATGACATCAACTTGTCAT	39.5
2036855.5	*yedW*	*-*	TCATTACAAAATTGTAAT	−53.5
3670466.5	*yhjC*	−0.7827	GTTTCACAATGTTGTCAT	−91.5
(B) Transportation				
**259612.5**	***phoE***	8.2168	**ATTTATTAAATCTGTAAT**	**−134.5**
594808.5	*cusC*	2.7525	AAATGACAAAATTGTCAT	−79.5
871560.5	*gsiD*	*-*	ACGCAACAAAACGGTCAT	626.5
1107869.5	*mdoC*	*-*	ACGTATTATATTTGTCAT	384.5
1575128.5	*yddB*	*-*	TTGACAGATAGTTGTCAT	1428.5
2136092.5	*yegH*	−0.9238	TTGTATGACAAATGTCAC	−30.5
2238620.5	*mglB*	*-*	GTATCTTAACAATGTGAT	−242.5
3029561.5	*ygfU*	*-*	TCGTCACATTATTGCAAT	234.5
**3590504.5**	***ugpB***	2.7377	**CTATCTTACAAATGTAAC**	**−105.5**
3609847.5	*yhhS*	*-*	ATGAAACACTGTTGTAAA	−24.5
3710399.5	*yhjX*	*-*	TTTTAACGTAACAGTCAC	−225.5
**3910006.5**	***pstS***	6.2008	**CTGTCATAAAACTGTCAT**	**−97.5**
4140311.5	*frwC*	*-*	ATGTGACAAATCTGCCAA	−239.5
4140311.5	*ptsA*	*-*	ATGTGACAAATCTGCCAA	−69.5
4225453.5	*yjbB*	*-*	GATTCACAAATCTGTCAC	−212.5
4323404.5	***phnC***	8.2648	AATTAACCAAATCGTCAC	−53.5
(C) Metabolism				
259612.5	*proB*	*-*	ATTTATTAAATCTGTAAT	−153.5
347564.5	*prpB*	*-*	TTGCAACAATTATGAAAC	−186.5
**400879.5**	***phoA***	6.0955	**CTGTCATAAAGTTGTCAC**	**−71.5**
416096.5	*sbcD*	−0.6432	TTTTCATAAATCTGTCAT	−117.5
466908.5	*cof*	0.9983	ATGTCAAAAAAATGCCAC	408.5
659845.5	*lipA*	*-*	TTGTCAAAATGTTGTAGC	−423.5
713691.5	*pgm*	*-*	GAGTGACGATACCGTGAC	933.5
791061.5	*galE*	*-*	TTACGACAACAATGTCAA	295.5
1369357.5	*ycjM*	*-*	GTATGAAATAAATGTGAC	1147.5
1445357.5	*feaB*	*-*	TTGCAACACAAATGCAAC	−192.5
1564180.5	*yddV*	0.6879	CTGTAATAAAAATTTCAC	803.5
1628303.5	*ydfI*	*-*	ATGCCAGAAAACGGTCAT	560.5
1654897.5	*speG*	*-*	GTAAAACAGAGCTGTGAA	682.5
1864699.5	*mipA*	2.0870	TTGCATAATTAATGTAAA	−180.5
1864699.5	*yeaG*	*-*	TTGCATAATTAATGTAAA	−255.5
2487014.5	*yfdE*	*-*	GATTAATAAAACTGTAAT	195.5
3029561.5	*ygfT*	*-*	TCGTCACATTATTGCAAT	−670.5
3079040.5	*tktA*	*-*	TAGAAATACCGTTGTCAT	555.5
3241621.5	*uxaA*	*-*	CTGTCATACACCCGTCAC	−170.5
3935233.5	*rbsK*	*-*	GTGACATAAATACGTCAT	−90.5
(D) Membrane protein				
2048951.5	*yeeJ*	*-*	CTGCCACATTAACGTCAT	6024.5
2741390.5	*yfiB*	*-*	CTGACACAAATCTTTAAT	−183.5
3360618.5	*yhcD*	*-*	CGGTAATAAATATGTCAC	−42.5
3609847.5	*yhhT*	*-*	ATGAAACACTGTTGTAAA	−107.5
(E) Unknown function				
1442537.5	*ydbH*	*-*	GCGTAATCGTGCTGTCAT	1383.5
1579652.5	*ydeN*	*-*	TTGTCAAAAATCAGTAAT	563.5
1654897.5	*ynfC*	*-*	GTAAAACAGAGCTGTGAA	590.5
2036855.5	*yedX*	1.5615	TCATTACAAAATTGTAAT	−79.5
(F) Pseudo gene				
4571938.5	b4486	-	CAGCGACAAAATTGTGAC	236.5

a Entries in bold indicate they are known binding targets listed in a previous study [9].

b The log_2_-ratios of differential gene expressions in our microarray analysis. Dash symbols indicate no differential expression.

c The distance from the center of the putative binding site to the translational start site. A positive value means the site is located in the CDS while a negative value indicates the site is in the upstream region of the corresponding gene.

### Previously uncharacterized PhoB binding targets

**Figure 2:**
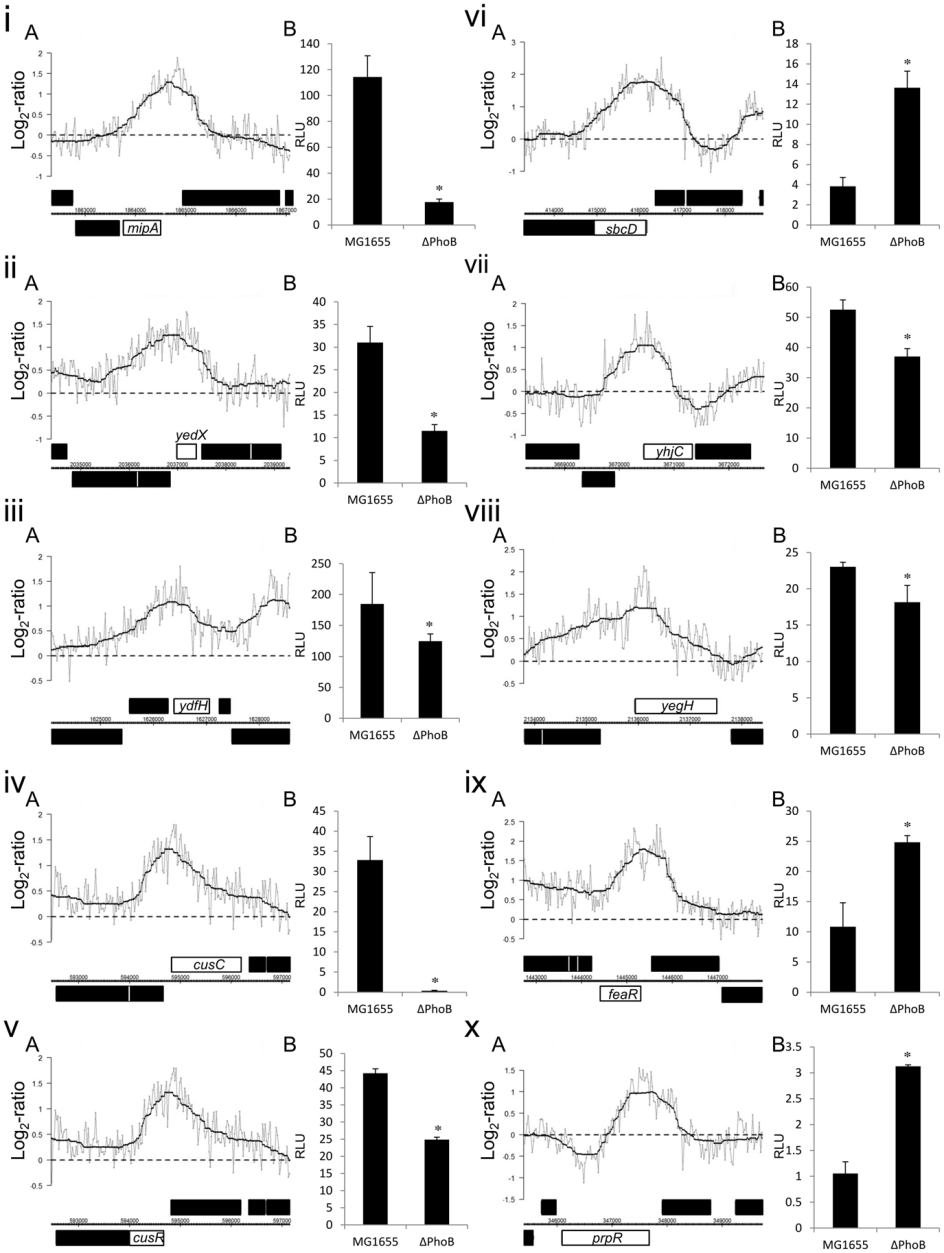
Novel findings of PhoB targets with differential expressions in the presence and the absence of PhoB activity. This figure shows the (A) ChIP-chip peaks and the (B) luminescence from reporter gene assay of PhoB novel targets which located in the promoter regions with differential expressions in MG1655 and MG1655_PhoB_KO. These targets are (i) *mipA*, (ii) *yedX*, (iii) *ydfH*, (iv) *cusC*, (v) *cusR*, (vi) *sbcD*, (vii) *yhjC*, (viii) *yegH*, (ix) *feaR*, and (x) *prpR*. (A) Expansion of PhoB binding peaks on the ten regions. The detected peaks were centered with 2000 bps flanking regions. The log_2_ fold change (*y*-axis) represents the log_2_ ratio (grey line) and the smoothed ratio (black line) of the normalized Cy5 signal (MG1655_PhoB_FLAG) divided by the normalized Cy3 signal (MG1655) after averaging our triplicate results. (B) Reporter gene assays were performed to further check the differential expression. Luminescence is expressed as relative light unit (RLU). The RLU was calculated by, first normalizing the light units to the optical densities of harvested cultures, then divided by the averaged light units from non-inserted pGL3 vector transformed MG1655 strain or MG1655_PhoB_KO strain (shown as ΔPhoB). The “*” sign indicates significant luciferase expression (p-value<0.05).

Eight novel PhoB binding sites are adjacent to ten genes that were shown to be differentially expressed in our analysis of gene expression microarray (see below). These ten genes are likely to be directly regulated by PhoB. Promoter regions containing these target sites were amplified and cloned into the promoterless luciferase expression vector pGL3 to create the promoter::luciferase fusions. These fusion plasmids were transformed into the *E. coli* wild type strain and the *phoB* knockout strain. We found that all ten plasmids showed significant differences in luciferase expression (Figure 2).

**Figure 3:**
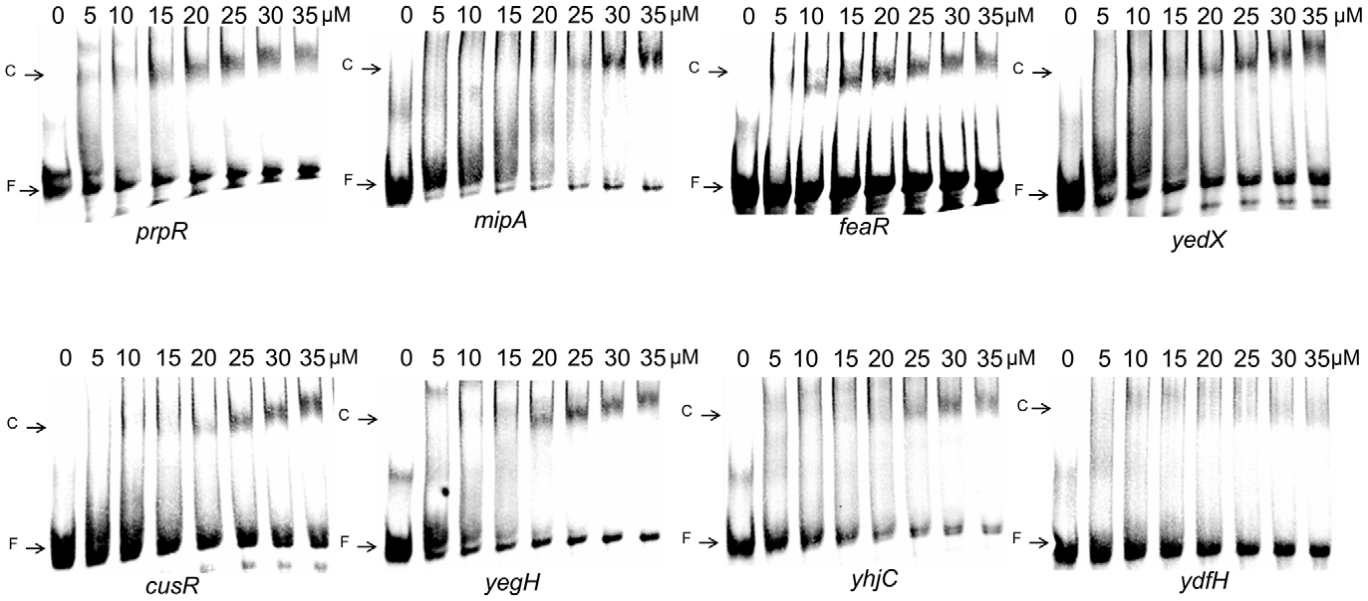
Binding of PhoB to its targets *in vitro*. This figure shows the results of gel mobility shift assays for binding of PhoB-His fusion protein to the eight binding sites. The binding sites which are centered at their putative binding sites corresponding to their intergenic regions upstream of the *prpR*, *mipA*, *feaR*, *yedX*, *cusR*, *yegH*, *yhjC*, and *ydfH* genes. DNA fragments were incubated with 0, 5, 10, 15, 20, 25, 30, or 35 μM PhoB-His as indicated. Free DNA fragments and PhoB-His-DNA complexes are labeled as F and C respectively.

These eight binding sites are related to the ten targets since there are two divergently transcribed gene pairs which share the same putative binding sites. To further examine if these eight sites are directly bound by PhoB, we used the gel mobility shift assay to detect the protein-DNA interactions. Synthesized single-stranded DNA fragments covering the putative binding sites were first end-labelled with biotin, annealed and incubated with the purified PhoB-His fusion protein *in vitro*. The purity of our PhoB-His fusion protein is shown in Figure S3. From the results of gel mobility shift assays, the two putative binding sites located upstream of *yhjC* and *ydfH* were seen as having low-affinity binding in our *in vitro* experimental conditions. The other six binding targets also showed different affinities for PhoB binding as the shifts occurred at different concentrations of PhoB (Figure 3).

### Identification of the PhoB binding pattern

The PhoB binding pattern can be identified using motif analysis of the enriched peaks from the ChIP-chip results. All 43 enriched regions were input into the MEME software to find conserved patterns. The most significant 18 bp pattern was identified (e-value  = 2.2e−18), meaning that all currently identified targets of PhoB share a significant conserved pattern (Table 2). The sequence logo representation of this pattern is shown in Figure S2C. This pattern clearly agrees with the known PhoB binding pattern (Figure S2D).

Surprisingly, nearly half (20/43) of the binding targets were located within the coding regions and this percentage is relatively higher than that of other transcriptional regulators mentioned in the study by Shimada *et* *al* [13]. They observed that the RutR regulator also has a high percentage of binding sites (90%) located in the coding regions. This could be due to incomplete evolution to eliminate the non-functional DNA sites or uncharacterized regulations. In contrast to RutR, PhoB is a well-conserved protein and the phosphate-sensing mechanism is vital for survival; thus PhoB is likely well-evolved and its bindings in the coding regions may have biological functions.

Differentially expressed genes containing putative PhoB binding sites in their coding regions were selected to confirm that PhoB binds to their coding regions and may participate in regulating them. Three genes: *cof*, *yahA*, and *yddV* (shown in Table 2), fit the criteria and the 60 bps centered at the three putative PhoB binding sites were further tested by gel mobility shift assays (Figure S4). Although the detailed mechanisms involved remain to be defined, the results here reveal that PhoB plays roles in the regulation of gene expression through binding to the coding regions.

### Functional categories altered by PhoB

To assess the gene expression status affected by PhoB, RNA samples were extracted from the MG1655_PhoB_KO and the MG1655 strains under the same condition used for the ChIP-chip experiments. Followed by cDNA synthesis, biotin-labelling and hybridization onto the Affymetrix array, gene expression status was measured. There were 287 differentially expressed genes that were directly or indirectly regulated by PhoB (Table S1). Within these 287 differentially expressed genes, 177 genes were up-regulated while 110 genes were down-regulated with PhoB activity.

**Figure 4:**
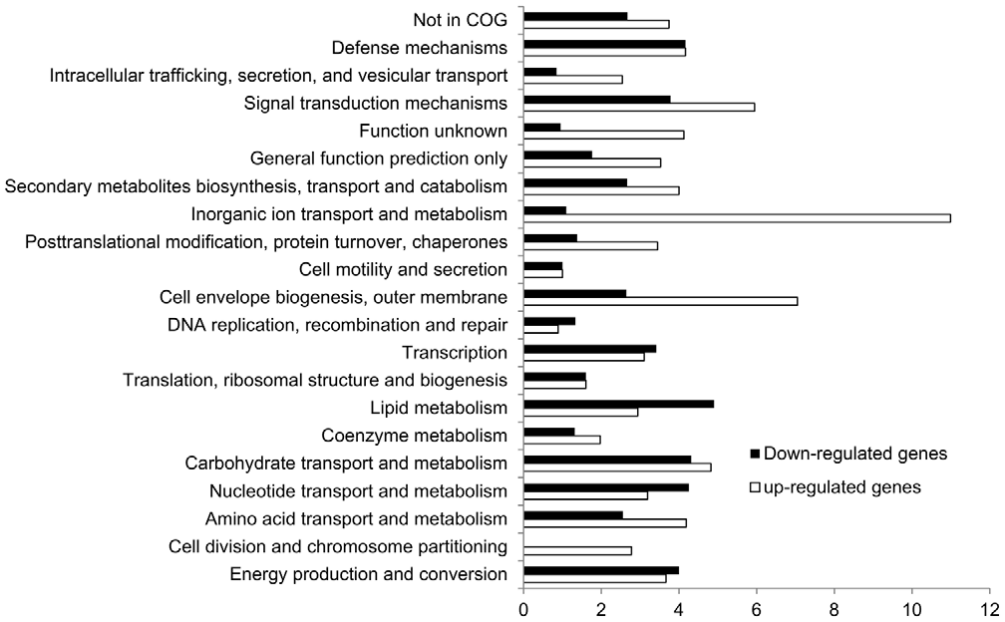
COG functional groups of differentially expressed genes. The white and black bars represent the distribution of 177 up-regulated genes and 110 down-regulated genes, respectively, in the phosphate sufficient conditions compared to the phosphate limiting condition. Genes annotated with multiple COG categories were placed into each assigned COG category.

In order to investigate the global biological roles played by PhoB under the phosphate-limiting condition, the COG functional distribution of these differentially expressed genes was plotted (Figure 4). It is reasonable to see that a large group (>10%) of genes participated in inorganic ion transportation and metabolism and was up-regulated during phosphate starvation in order to enhance phosphate uptake and usage. Additionally, about 7% of genes participating in cell envelope biogenesis/outer membrane were also up-regulated.

### The hierarchical structure of phosphate sensing regulatory network

It is worth noting that our gene expression data showed 287 genes affected by PhoB while only 19 out of these 287 genes were considered to be directly regulated PhoB targets (Table 2). There are 22 differentially expressed transcriptional regulators and six of them (*cusR*, *feaR*, *phoB*, *prpR*, *ydfH*, and *yhjC*) contained the Pho box within their upstream regions. Thus PhoB may pass the phosphate-limiting signal first to the six regulators which they then regulate the other 15 regulators, which in turn affect the remaining 265 differentially expressed genes. Under the hierarchical structures of phosphate signalling passages, feed forward loop (FFL) network motifs play a role in signal sensing and responding mechanisms. Each FFL network motif contains three genes. Two of the three are transcription regulators, one of which regulates the other, and they jointly regulate the third target gene. At least four sets of gene pairs, *cusR*/*cusC*, *prpR*/*prpB*, *feaR*/*feaB*, and *yedW*/*yedX*, have the potential to form feed forward loops with PhoB regulation. For example, PhoB regulates *cusR* and both PhoB and CusR regulators regulate *cusC*. Thus, the *phoB*, *cusR*, and *cusC* form a FFL network motif. A previous study has demonstrated *in silico* that these FFLs can enhance the signal transduction processes or delay the response or adjust the sensing mechanisms through transcriptional regulation [14]. The underlying biological functions of these potential FFLs are left for future *in vivo* investigations. Overall, the data suggest that PhoB specifically regulates a relatively small group of genes, which influence a large group of downstream genes during phosphate starvation.

### PhoB operates cooperative regulatory mechanisms

In addition to the formation of FFL network motifs, three more observations revealed that PhoB cooperates with other transcription factors to modulate downstream responses. The first one is the contrasting regulatory modes observed from our reporter gene assay and microarray data. The two upstream regions of *yhjC* and *yegH* may be positively regulated by PhoB based on our reporter gene assay. However, they both showed down regulation in the presence of PhoB from our microarray datasets. The opposite results indicate that other regulators may have inhibitory roles in the coding regions to block the up-regulation of PhoB in our experimental condition. In addition, post-transcriptional modifications, such as mRNA degradation or small RNA regulation, may also be other reasons for reductions in PhoB up-regulation. Secondly, only about 35% of PhoB binding targets showed differential expression (Table 2). This may be a clue that PhoB cooperates with other factors to modulate transcription since other factors may reduce the effect of PhoB regulation. Finally, we also observed an indirectly regulated 14 kb region to which PhoB did not tend to bind to (Figure S5). It is interesting that genes in this 14 kb region all showed up-regulation in the presence of PhoB. This result suggests that other factors may protect this highly expressed region from PhoB binding. Although the underlying mechanisms require further investigation, these observations implicate that PhoB operates complex regulatory mechanisms and cooperates with other factors in the signal integration of genetic regulatory networks in *E. coli*.

### PhoB is involved in the regulation of transporter systems and membrane component rearrangement

Out of the 287 genes showing differential expression in our gene expression experiments, more than 60 genes encode the proteins of transporter systems (Table S1). Previous reports had shown that phosphorus-uptake related transporters are activated during phosphate starvation [1]. Based on our results, a large group of genes encoding transporter systems were also activated, such as: Oligopeptide transporter (*oppABCDF*), Copper/silver efflux system (*cusCFBA*), multidrug-efflux systems (*mdtABCD*, *cmr*), neutral amino-acid efflux system (*eamB*) and others (Table S1). These transporters may play roles to adjust the overall metabolic flux of the cells although the adjustments involved are not clear at this time.

Membrane constituents such as lipopolysaccharides, outer-membrane proteins, and membrane lipids have been reported to be regulated during phosphate starvation [15], [16]. This may be because phosphorus is a major component of cell membrane-forming phospholipids. If the phospholipids cannot be renewed, the membrane becomes too weak to defend stresses like oxidation pressure, osmotic stress, and others. Our microarray data showed that a group of genes related to the metabolisms of murein, palmitoylated lipid A, colanic acid, and putrescine are modulated under PhoB activity.

### Murein

Murein or peptidoglycan can help *E. coli* cells to stabilize their cell envelope under the high intracellular pressure [17]. The genes *mipA* (scaffold protein for murein synthesizing machinery), *ycfS* (L, D-transpeptidase linking Lpp to murein) and *mltD* (predicted membrane-bound lytic murein transglycosylase) were observed to be activated in transcriptional expression. The *mipA* gene also has a PhoB binding signal in its upstream region and is considered as a directly regulated target.

### Lipid A

For the modification of lipid A, the hexa-acyl pyrophosphate Lipid A is known to be modulated through the Pho regulon in *E. coli* [18]. From our microarray data, we observed the involvement of PhoB in the up-regulation of *pagP*. Palmitoylated lipid A may also be synthesized during phosphate starvation since PagP transfers palmitate from phospholipid to lipid A precursor to generate palmitoylated lipid A, which protects bacteria from host defences and is likely related to bacterial virulence [19], [20].

### Colanic acid

Additionally, colanic acid is an extracellular polysaccharide and has been shown to increase tolerance to heat and acid conditions [21], [22]. The genes, *wzxC* (colanic acid exporter), *wcaJ* (predicted UDP-glucose lipid carrier transferase), *wcaK* (predicted pyruvyl transferase), *wcaL* (predicted glycosyl transferase) and *wcaM* (predicted colanic acid biosynthesis protein) are involved in the colanic acid biosynthesis and transportation pathway and were observed to be up-regulated in the wild-type strain relative to the PhoB knock-out strain.

### Putrescine

As for the linear polyamine, putrescine, its role is related to membrane stabilization and optimal growth. However, a high concentration of polyamines will inhibit cell growth and protein synthesis. Therefore, the polyamine degradation pathway exists in bacteria for balancing the concentration [23]. This pathway involves *puuCBE* (gamma-Glu-gamma-aminobutyraldehyde dehydrogenase, gamma-Glu-putrescine oxidase and GABA aminotransferase), *puuP* (putrescine importer), *puuA* (gamma-Glu-putrescine synthase), and *puuD* (gamma-Glu-GABA hydrolase). In our study, all of these genes showed down-regulation and their transcription repressor, PuuR, in turn was up-regulated in the presence of PhoB activity. The repressed putrescine degradation pathway indicates that, during phosphate starvation, membrane stabilization is more important than growth since *E. coli* cells enter the stationary phase.

### PhoB is involved in oxidative stress protection

Previous studies described that although cells stop growing, bacteria will still undergo aerobic respiration during phosphate starvation [24]. Under this circumstance, hydrogen peroxide may not be diluted through cell division and thus may accumulate in cells. Oxidative stress was demonstrated to occur during phosphate starvation. In addition, the alkyl hydroperoxide reductase (AHP) complex helps scavenge hydrogen peroxide produced during phosphate starvation [25], [26]. In our study, the *ahpCF* was identified to be up-regulated indirectly by PhoB. This suggests that PhoB plays a protective role for the oxidative stress which occurs during phosphate starvation.

It is known that methylglyoxal is synthesized to enhance the phosphate turnover during phosphate starvation [27], [28]. Although methylglyoxal can help to protect against electrophile attack and detoxification, excess methylglyoxal leads to cell death. From our gene expression analysis, the *yeaE* gene encoding the methylglyoxal reductase was up-regulated in the wild-type *E. coli* strain compared to the PhoB knock-out strain. This is another indication that PhoB has a protective role for oxidative stress produced during phosphate starvation.

### PhoB participates in protecting cells during phosphate starvation

We have presented that during phosphate starvation, PhoB is involved in triggering the membrane component rearrangement for membrane integrity. In addition, PhoB also indirectly affects genes participating in protecting cells from oxidative stress and genes that balance the level of methylglyoxal. These results together suggest that PhoB protects the bacterium by enhancing membrane integrity and reducing oxidative damage to the cell membranes. We have identified several predicted transcription factors that are regulated by PhoB. Further studies of these predicted transcription factors are needed in order to understand the complex interplay between genes and regulators in the bacterial signalling and regulatory networks during phosphate starvation.

In summary, our genome-wide approach for characterizing the roles of PhoB by ChIP-chip and gene expression array provides a comprehensive global binding profile of PhoB. We have presented a hierarchical structure of transcriptional regulators of the phosphate-sensing network as well as the potential membrane protective roles of PhoB.

## Materials and Methods

### Bacterial strains, plasmids and growth conditions

Bacterial strains used in this study are shown in Table 1. Tables S2 and S3 list the plasmids and the oligonucleotides, respectively. A *phoB* knock-out derivative from the BW25113 strain was requested from Keio collection [29]. This *phoB* disruption was then transferred into MG1655 strain by P1 transduction [30] and named MG1655_PhoB_KO. The MG1655_PhoB_FLAG which carries a 3xFLAG tag at the 3′ end of *phoB* gene was constructed from the BW25113 strain using an epitope tagging approach [31].

For PhoB ChIP-chip experiments, strains MG1655 and MG1655_PhoB_FLAG were grown in Morpholinepropanesulfonic acid (MOPS) minimal medium with 200 μM K_2_HPO_4_ and 0.4% glucose. Figure 1 shows the time point for cell harvesting and the cultivation of MG1655, MG1655_PhoB_FLAG, and MG1655_PhoB_KO under phosphate-limiting and phosphate-sufficient conditions. The time point at OD_600nm_ of 1.0 was selected since phosphate was used up and PhoB had a higher activity for ChIP-chip assay. For the gene expression microarray experiments, MG1655 and MG1655_PhoB_KO were compared under the same conditions as the ChIP-chip assay. To compare the promoter activity of the upstream regions, promoter::luciferase gene fusion plasmids were constructed, and luminescence was measured for both MG1655 and MG1655_PhoB_KO strains at the same time point as the two above experiments (Figure 1). The ChIP-chip experiments and the reporter gene assays were carried out in at least biological triplicates, while the gene expression microarray experiments were performed in two biological replicates.

### Determination of phosphate concentration

To determine the concentration of orthophosphate, an ascorbic acid method described previously was applied in biological triplicates with slight modifications [32]. After overnight culturing of the MG1655, MG1655_PhoB_KO, and MG1655_PhoB_FLAG strains in MOPS minimal medium containing 1000 μM K_2_HPO_4_ and 0.4% glucose, cultures were diluted in 1∶100 ratio in MOPS minimal medium containing 200/1000 μM K_2_HPO_4_ and 0.4% glucose and grew at 37°C. At each time point, cultures were collected, centrifuged at 12,000 g for 5 min, and then 1 ml supernatants were added to 160 μl reaction solution (1 N sulphuric acid, 0.1 mM potassium antimonyl tartrate, 4.8 mM ammonium molybdate and 30 mM ascorbic acid (added lastly)). After 10 min incubation of supernatants with the reaction solution, the light absorbance at 880 nm was measured. By interpolation of the standard curve, the phosphate concentration was determined.

### Chromatin immunoprecipitation (ChIP) experiment

To identify the genome-wide DNA-binding profile of PhoB, ChIP assays were performed on MG1655_PhoB_FLAG and MG1655. The ChIP assay protocol was modified from Byung-Kwan Cho *et* *al*. [33]. The MG1655_PhoB_FLAG strain expresses the PhoB-FLAG fusion protein where the FLAG tag can be recognized by anti-FLAG antibody and used for ChIP assaying [34]. The MG1655 strain, which expresses no FLAG tag, was used as a control group. Cultures were grown to an OD_600_ value of 1.0 and treated with 1% formaldehyde for 10 min. To quench the reaction, glycine was added at the final concentration of 0.125 M for 5 min. Cells were centrifuged at 12,000 g at 4°C for 20 min and washed two times with the washing buffer (10 mM Tris-HCl (pH 7.4), 0.1 M NaCl, 1 mM EDTA and 0.5% Tween-20). The washed cells were then lysed with the lysis buffer (10 mM Tris-HCl (pH 7.4), 0.1 M NaCl, 1 mM EDTA and 0.5% Tween-20, 8 kU/ml lysozyme, 1 mM PMSF, and protease inhibitor cocktail (Sigma)) for 30 min at 4°C. The lysates were sonicated (Bioruptor) to result in DNA fragments ranging from 100 bps to 1000 bps with the average size of 500 bps. After sonication, the lysates were centrifuged at 12,000 g for 20 min at 4°C and the resulting supernatants were used for immunoprecipitation.

To eliminate the non-specific bindings between the magnetic beads coated with Protein G (Invitrogen) and the anti-FLAG antibody, the magnetic beads were pre-incubated with 0.05 mg/ml anti-FLAG antibody (Sigma). Similarly, for the purpose of eliminating the non-specific bindings between our lysates and the beads, lysates were also pre-cleared by incubating them with the beads without the anti-FLAG antibody. To immunoprecipitate the PhoB-FLAG-DNA complex, beads pre-incubated with the antibody were added in both lysates from MG1655_PhoB_FLAG and MG1655 strains at 4°C overnight. The beads were washed once with IP buffer (10 mM Tris-HCl (pH 7.4), 0.1 M NaCl, 1 mM EDTA, and 0.05% [v/v] Tween-20 and 1 mM fresh PMSF), twice with ChIP wash buffer I (10 mM Tris HCl (pH 7.4), 300 mM NaCl, 1 mM EDTA, 0.1% Tween-20 and 1 mM fresh PMSF), three times with ChIP wash buffer II (10 mM Tris-HCl (pH 7.4), 500 mM NaCl, 1 mM EDTA, 0.1% [v/v] Tween-20 and 1 mM fresh PMSF), once with ChIP wash buffer III (10 mM Tris-HCl (pH 7.4), 250 mM LiCl, 1 mM EDTA, 0.1% [v/v] Tween-20 and 1 mM fresh PMSF) and once with TE buffer (10 mM Tris-HCl (pH 7.4) and 1 mM EDTA). After removing the TE buffer, beads were incubated twice with elution buffer (50 mM Tris-HCl (pH 7.4), 10 mM EDTA and 1% SDS) at 65°C for 15 min and the two resulting eluted solutions were combined.

After incubating the combined eluted samples with proteinase K (Sigma) to the final concentration of 10.5 U/ml at 42°C for 2 hours, the reverse cross-link procedure was performed by incubating at 65°C overnight to unlink the covalent bonds formed by formaldehyde between peptides and DNA. Samples were then treated with RNase A (Sigma) to the final concentration of 26 μg/ml, followed by purifying DNA from the RNase A-treated samples using the PCR purification kit (Qiagen).

### Whole genome tiling array analysis for ChIP-chip experiments

The NimbleGen 385 K high density tiling array for *E. coli* K12 MG1655 (Cat. No. 05542901001) was used for our ChIP-chip assay. The instructions of the NimbleGen's protocol (version 2.0) were followed for all procedures. Immunoprecipitated samples were amplified by whole genome amplification kit (Sigma) twice and pooled together. The amplified samples from MG1655_PhoB_FLAG were labelled with Cy5 dye while the control samples from MG1655 were labelled with Cy3 dye. After the hybridization step, the arrays were washed and then scanned with an Axon scanner (GenePix 4000B).

The scanned TIF image files were then processed by NimbleScan software to generate the intensity pair files. The R package Ringo [35] was used to read the pair files, and the limma package [36] was used for within- and between-array normalization [37]. The averaged values of normalized Cy5 and Cy3 intensities from triplicate samples were used to calculate the log_2_-ratios (Cy5/Cy3). The enriched regions were then identified by the CMARRT package [12] with a controlled error rate set at 0.05. Our ChIP-chip data had been submitted to NCBI GEO database and the GSE Series record is GSE21857.

### Motif identification

To find the position weight matrix (PWM) of PhoB binding sites, *E. coli* K12 MG1655 sequences of all the enriched regions were extracted from NCBI RefSeq (accession no. NC_000913). We used the MEME program [38] to search for the most significant conserved pattern with pattern length ranging from 18 to 22 bps (accomplished by using the –minw 18–maxw 22 options of MEME). The range was selected because the previously reported PhoB binding pattern is 18 bps in length [11], while the structure information indicated that the site is 22 bps [10]. A seven-order background model was built from the whole *E. coli* K12 MG1655 reference sequence (accomplished by using the –bfile <background model file> option in MEME). In addition, sites on both strands were allowed (accomplished by using the –revcomp option). The sequence logo [39] was then used to present the PWM graphically.

### Gene expression microarray and analysis

The Affymetrix *E. coli* Genome 2.0 array was used to investigate gene expression status in the presence and the absence of PhoB activity. The *E. coli* K12 MG1655 and MG1655_PhoB_KO strains grew in MOPS minimal medium containing 200 μM K_2_HPO_4_ and 0.4% glucose. At an OD_600_ of 1.0, cultures were treated with 10 mg/ml lysozyme and 10% SDS at 4°C for 5 min to lyse bacterial cells. Then, the protocol for total RNA purification using TRIZOL reagent (Sigma) was followed. The Affymetrix standard protocol was then applied for cDNA synthesis, fragmentation, biotin labelling and hybridization. The raw CEL files were normalized by a robust multi-array average approach [40]. The microarray data have also been included in GSE21857 of NCBI GEO database. To assess statistically significant differential expression, we applied linear models and empirical Bayes methods [41] through the limma package, and the Benjamini and Hochberg's q-value threshold was set at 0.05. The filtered results were considered as the differentially expressed genes. To investigate the functions of the differentially expressed gene, the functional categories of the clusters of orthologous group (COG) were used [42].

### Construction and assay of promoter::luciferase fusions

The promoter regions of the PhoB targets identified in ChIP-chip experiments were amplified by PCR from the MG1655 strain using the primers listed in Table S3. After treatment with NheI and NcoI restriction enzymes, the digested linear products were then ligated into a NheI-NcoI digested pGL3-basic vector (Promega). The pGL3 plasmid contains a promoterless luciferase gene. The cultivation condition was in MOPS minimal medium supplemented with 200 μM K_2_HPO_4_ and 0.4% glucose at the same condition as the experiments for ChIP-chip assay and gene expression microarray (Figure 1). The luciferase activities were measured using a luciferase assay system (Promega).

### PhoB-His fusion protein purification

In order to overexpress the PhoB-His fusion protein, the PhoB coding region was cloned into a pET21d (+) plasmid. This constructed vector expressing the PhoB-His_(6x)_ fusion protein was transformed into BL21. Overnight cultures were diluted 1∶500 into 250 mL LB cultures containing 100 μg/μl ampicillin. The cultures were grown at 37°C until an OD_600_ of 0.4∼0.6, then treated with 1 mM IPTG to induce PhoB-His_(6x)_ expression and then grown at 37°C for another 2 hours. After centrifugation, the pellets were resuspended in 10 ml lysis buffer (20 mM NaH_2_PO_4_, 500 mM NaCl, 20 mM imidazole, and 1 mg/ml lysozyme). The cells were lysed for 30 min at 4°C and then the lysates were cleared by centrifugation at 14000 g for 30 min at 4°C. After applying the lysate to the Ni-sepharose column (GE Healthcare), the column was washed two times by 4 ml wash buffer (20 mM NaH_2_PO_4_, 500 mM NaCl, and 30 mM imidazole). The elution was performed by applying 1 ml elution buffer (20 mM NaH_2_PO_4_, 500 mM NaCl, 500 mM imidazole) to the column four times. The eluted samples were dialyzed in the storage buffer (25 mM Tris-HCl, 50 mM NaCl, 0.1 mM EDTA, and 0.1 mM DTT (pH 7.4)). The concentration of PhoB-His fusion protein was determined by the Bradford assay (Bio-Rad) using the bovine serum albumin (BSA) as the standard.

### Gel mobility shift experiments

The synthetic single-stranded 60 bp DNA fragments centered at PhoB putative binding sites were used in these experiments (Table S3). DNA fragments were first 3′-end labeled with biotin using a DNA 3′ End Biotinylation Kit (Pierce) and then annealed before use. Before the binding assay, the PhoB-His fusion protein was phosphorylated in the reaction buffer (50 mM Tris-HCl, 10 mM MgCl_2_, 0.1 mM DTT, and 20 mM acetylphosphate) at 37°C for 75 min [43]. The phosphorylated PhoB-His fusion protein was then used in the mobility shift assays. Each binding reaction contained 20 fmol 3′-end biotin labeled dsDNA, 20 mM Tris-HCl (pH 7.0), 50 mM NaCl, 1 mM DTT, 10 mM MgCl_2_, 100 μg/ml BSA, and 0.5 μg/ml poly dI-dC with various amounts of PhoB-His_(6x)_ fusion protein (see Figures 3 and S4). Reactions were incubated for 15 min at 37°C, and then loaded onto a 6% native polyacrylamide gel running at 100 V in 0.5X TBE buffer. After separation, samples were blotted to Amersham Hybond-N membranes using a Hoefer TE 70 device. The labeled biotin signals were transferred and detected using a LightShift Chemiluminescent EMSA Kit (Pierce) according to the manufacturer's instructions. For each tested target, at least two to three biological replicates were performed and the best figure was picked and shown in Figures 3 and S4.

## Supporting Information

Figure S1 **PhoB-FLAG fusion protein in MG1655_PhoB_FLAG strain has the same activity as PhoB in MG1655 wild type strain.** In order to confirm that the activity of our PhoB-FLAG fusion protein is not affected by the C-terminal FLAG tag, a reporter gene assay was used to measure the activity of the self-regulated PhoB promoter. The constructed *phoB* promoter::luciferase fusion plasmid were transformed into three strains, MG1655, MG1655_PhoB_FLAG, and MG1655_PhoB_KO (see Materials and Methods). The transformed strains were grown in MOPS minimal medium containing 0.2 mM K_2_HPO_4_ and 0.4% glucose until OD_600_ of 1.0. The growth condition and the time point are the same as our ChIP-chip assay and gene expression microarray. The luciferase activities were measured using a luciferase assay system (Promega). The y-axis in this figure shows the relative light unit (RLU). The *phoB*-deprived strain, MG1655_PhoB_KO, shows the basal level activity of *phoB* promoter without PhoB. The wild type MG1655 strain represents the activity of *phoB* promoter under wild type PhoB positive regulation. This figure displays that our PhoB-FLAG fusion protein in the MG1655_PhoB_FLAG strain has the same activity as the wild type PhoB protein.
(TIF)

Figure S2 **The genome-wide profile of PhoB binding regions across the** ***E. coli*** **genome.** (A) An overview of the results from ChIP-chip experiments at an OD_600_ value of 1.0. The log_2_ fold change (*y*-axis) is the log_2_ ratio (grey line) of the normalized Cy5 signal (MG1655_PhoB_FLAG) divided by the normalized Cy3 signal (MG1655) after averaging our triplicate results. These ratios were plotted against their locations on the 4.64 Mb *E. coli* chromosome (*x*-axis). (B) Expansion of PhoB binding peaks on the previously known regulatory sites. The detected peaks were centered with 5000 bps flanking regions and these peaks were located in the promoter regions of (i) *phoE*, (ii) *phoB*, (iii) *ugpB*, (iv) *pstS*, (v) *phoA*, and (vi) *phnC*. The log_2_-ratios (grey line) and the smoothed ratios (black line) on *y*-axis were calculated from the normalized Cy5 signal (MG1655_PhoB_FLAG) divided by the normalized Cy3 signal (MG1655) after averaging the triplicate results. (C) The most significant pattern was found in the 43 PhoB ChIP-chip peaks. The DNA sequences from all 43 PhoB ChIP-chip peaks (see Table 2) were combined and analyzed using the MEME program. This pattern was identified with the significant value of 2.2e−18 and then a sequence logo representation was generated by an R package called seqLogo. (D) The previously known PhoB binding pattern was retrieved from RegulonDB (http://regulondb.ccg.unam.mx/MatrixAlignment/results/). The first five bases of the known pattern was trimmed to produce an 18 bp pattern that can be compared with our pattern. Panels C and D in this figure show high similarity between these two patterns.
(TIF)

Figure S3 **Purity data for purification of PhoB-His fusion protein.** This figure displays the (A) SDS-PAGE and (B) the western blot results to demonstrate the purity of our purified PhoB-His fusion protein. The lanes from left to right are Marker (M), cell lysate (CL), flow-through (FT), the first washed fraction (W1), the second washed fraction (W2), the first eluted fraction (E1), the second eluted fraction (E2), the third eluted fraction (E3), the forth eluted fraction (E4), and the pooled and enriched fraction (P). The expected size of PhoB-His fusion protein is 27.9 kDa.
(TIF)

Figure S4 **Novel PhoB bindings located in the coding regions may participate in gene regulation.** The three genes, *yahA* (i), *cof* (ii), and *yddV* (iii), were shown to be differentially expressed in our gene expression microarray analysis. From our ChIP-chip analysis, significant PhoB binding peaks were detected and the putative PhoB binding motifs were also identified within the coding regions (panel A). For further investigation of PhoB bindings, the *in vitro* binding assays were carried out by gel mobility shift assay (panel B, see Methods for details). The results demonstrate that PhoB binds to the coding regions of the three genes despite the weak binding to *yahA*.
(TIF)

Figure S5 **A non-preferred binding region of PhoB.** In addition to PhoB binding regions, there is a long region in which the PhoB binding signals are lower than background noises. This region ranges from the genomic location of 1292000 to 1306000 bps. The y-axis represents the log_2_ ratio (grey line) and the smoothed ratio (black line) of the normalized Cy5 signal (MG1655_PhoB_FLAG) divided by the normalized Cy3 signal (MG1655) after averaging our triplicate results. In order to show the boundary of this non-preferred binding region, the genomic region from 1291000 to 1207000 bps is plotted. Genes located in this region are shown at the bottom of the plot. The pseudogene, *insZ*, located between *tdk* and *adhE* is not shown. All genes, *tdk*, *insZ*, *adhE*, *ychE*, *oppABCDF* and *yciU* were up-regulated with PhoB activity.
(TIF)

Table S1 **List of differentially expressed genes identified from the microarray analysis.**
(DOCX)

Table S2 **List of plasmids used in this study.**
(DOC)

Table S3 **List of oligonucleotides used in this study.**
(DOC)
