# Supplementary figures and images for: Genome-Wide PhoB Binding and Gene Expression Profiles Reveal the Hierarchical Gene Regulatory Network of Phosphate Starvation in Escherichia coli
Source: PLoS One. 2012 Oct 5;7(10):e47314. doi: 10.1371/journal.pone.0047314 (PMC3465305; doi:10.1371/journal.pone.0047314)

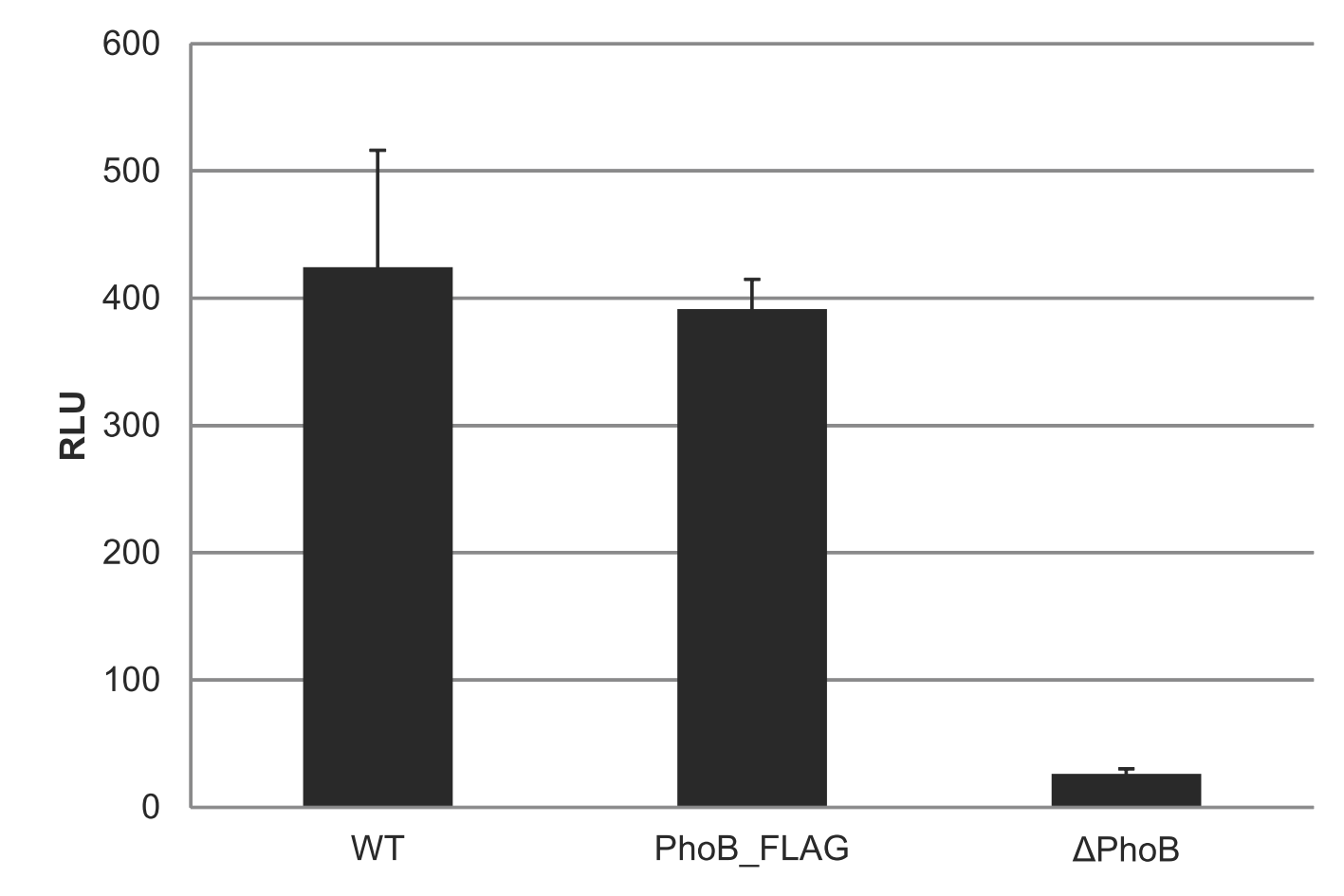

Supplement: Figure S1 — PhoB-FLAG fusion protein in MG1655_PhoB_FLAG strain has the same activity as PhoB in MG1655 wild type strain. In order to confirm that the activity of our PhoB-FLAG fusion protein is not affected by the C-terminal FLAG tag, a reporter gene assay was used to measure the activity of the self-regulated PhoB promoter. The constructed phoB promoter::luciferase fusion plasmid were transformed into three strains, MG1655, MG1655_PhoB_FLAG, and MG1655_PhoB_KO (see Materials and Methods). The transformed strains were grown in MOPS minimal medium containing 0.2 mM K2HPO4 and 0.4% glucose until OD600 of 1.0. The growth condition and the time point are the same as our ChIP-chip assay and gene expression microarray. The luciferase activities were measured using a luciferase assay system (Promega). The y-axis in this figure shows the relative light unit (RLU). The phoB-deprived strain, MG1655_PhoB_KO, shows the basal level activity of phoB promoter without PhoB. The wild type MG1655 strain represents the activity of phoB promoter under wild type PhoB positive regulation. This figure displays that our PhoB-FLAG fusion protein in the MG1655_PhoB_FLAG strain has the same activity as the wild type PhoB protein. (TIF) [file pone.0047314.s001.tif]

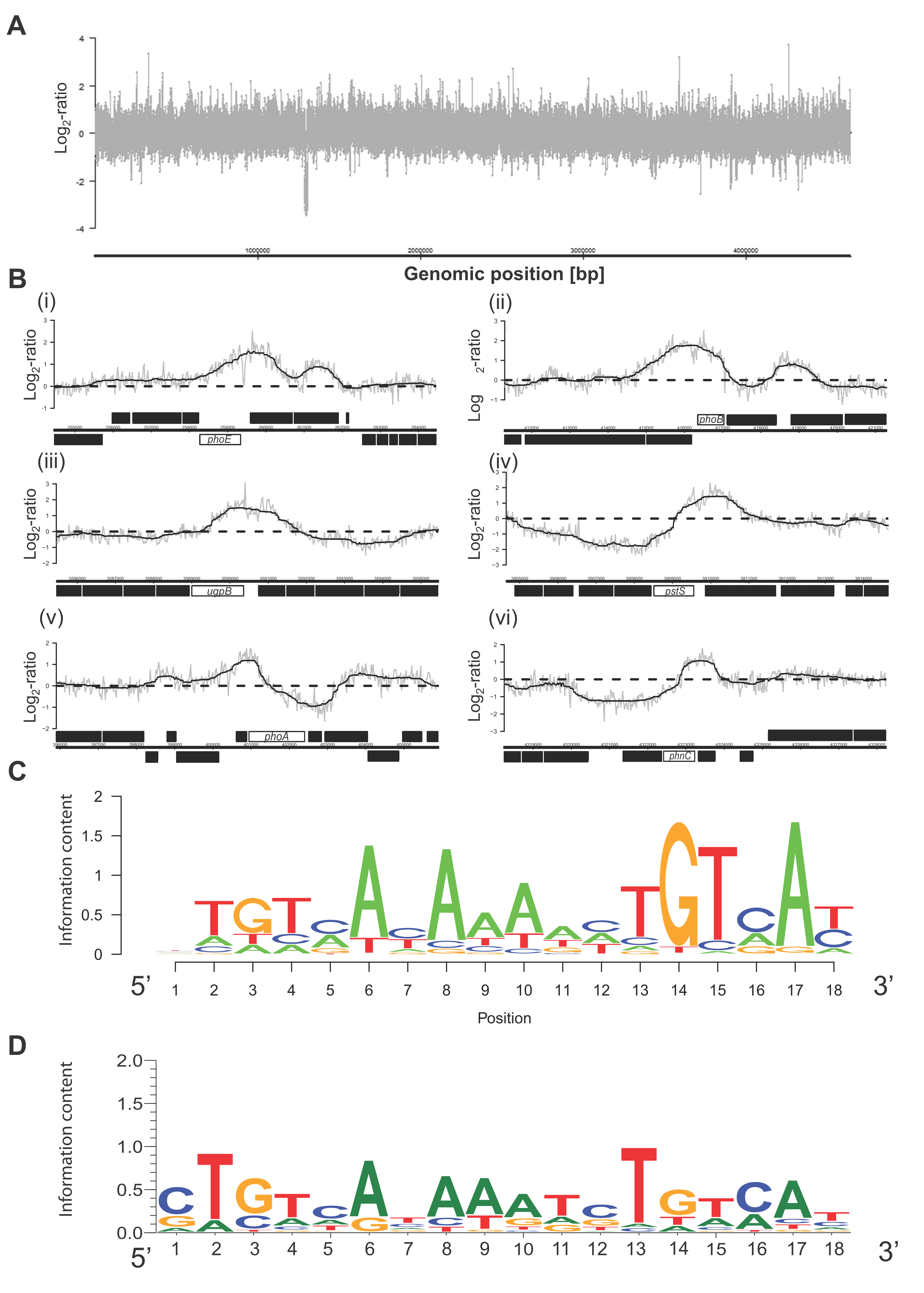

Supplement: Figure S2 — The genome-wide profile of PhoB binding regions across the E. coli genome. (A) An overview of the results from ChIP-chip experiments at an OD600 value of 1.0. The log2 fold change (y-axis) is the log2 ratio (grey line) of the normalized Cy5 signal (MG1655_PhoB_FLAG) divided by the normalized Cy3 signal (MG1655) after averaging our triplicate results. These ratios were plotted against their locations on the 4.64 Mb E. coli chromosome (x-axis). (B) Expansion of PhoB binding peaks on the previously known regulatory sites. The detected peaks were centered with 5000 bps flanking regions and these peaks were located in the promoter regions of (i) phoE, (ii) phoB, (iii) ugpB, (iv) pstS, (v) phoA, and (vi) phnC. The log2-ratios (grey line) and the smoothed ratios (black line) on y-axis were calculated from the normalized Cy5 signal (MG1655_PhoB_FLAG) divided by the normalized Cy3 signal (MG1655) after averaging the triplicate results. (C) The most significant pattern was found in the 43 PhoB ChIP-chip peaks. The DNA sequences from all 43 PhoB ChIP-chip peaks (see Table 2) were combined and analyzed using the MEME program. This pattern was identified with the significant value of 2.2e−18 and then a sequence logo representation was generated by an R package called seqLogo. (D) The previously known PhoB binding pattern was retrieved from RegulonDB (http://regulondb.ccg.unam.mx/MatrixAlignment/results/). The first five bases of the known pattern was trimmed to produce an 18 bp pattern that can be compared with our pattern. Panels C and D in this figure show high similarity between these two patterns. (TIF) [file pone.0047314.s002.tif]

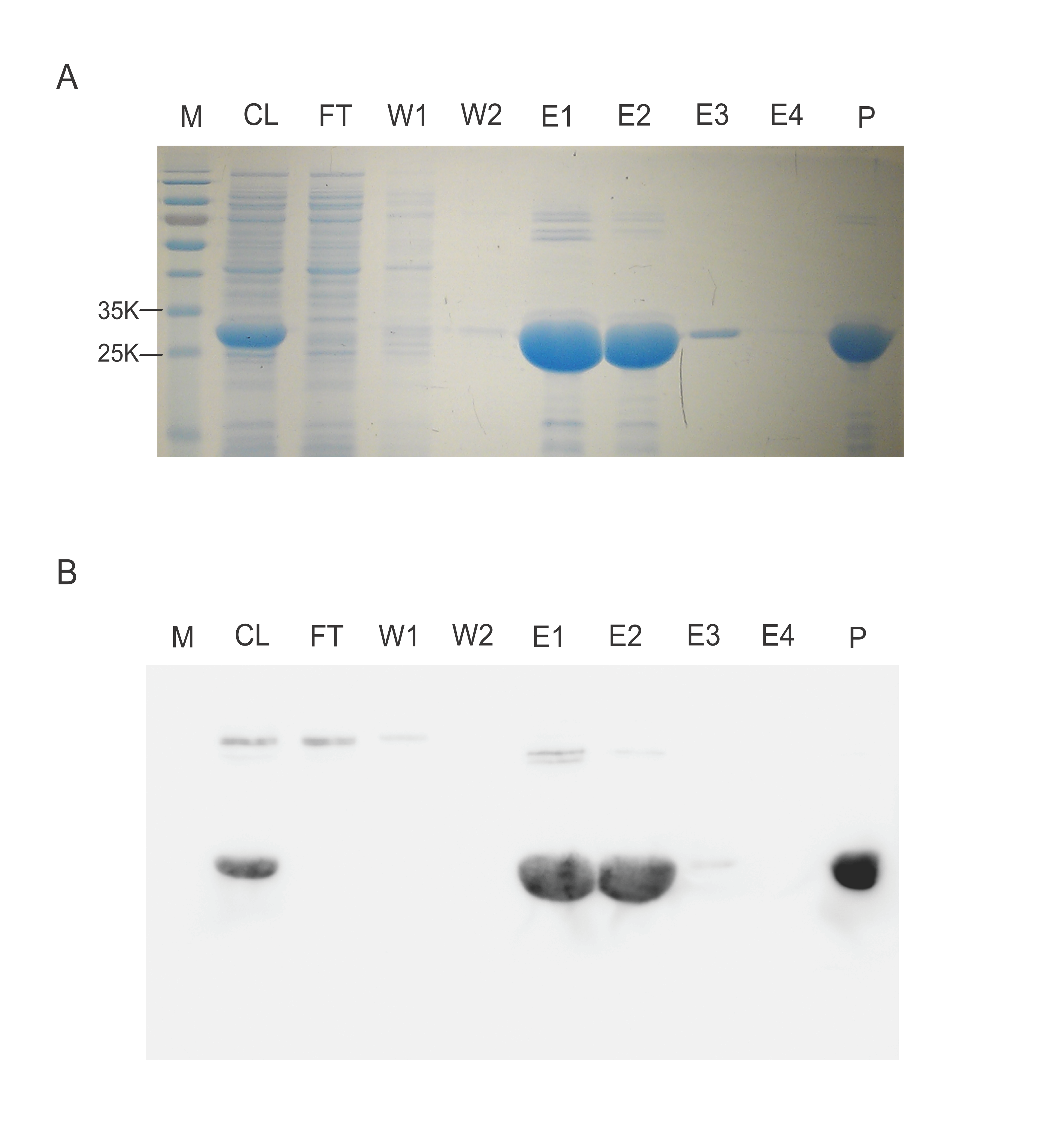

Supplement: Figure S3 — Purity data for purification of PhoB-His fusion protein. This figure displays the (A) SDS-PAGE and (B) the western blot results to demonstrate the purity of our purified PhoB-His fusion protein. The lanes from left to right are Marker (M), cell lysate (CL), flow-through (FT), the first washed fraction (W1), the second washed fraction (W2), the first eluted fraction (E1), the second eluted fraction (E2), the third eluted fraction (E3), the forth eluted fraction (E4), and the pooled and enriched fraction (P). The expected size of PhoB-His fusion protein is 27.9 kDa. (TIF) [file pone.0047314.s003.tif]

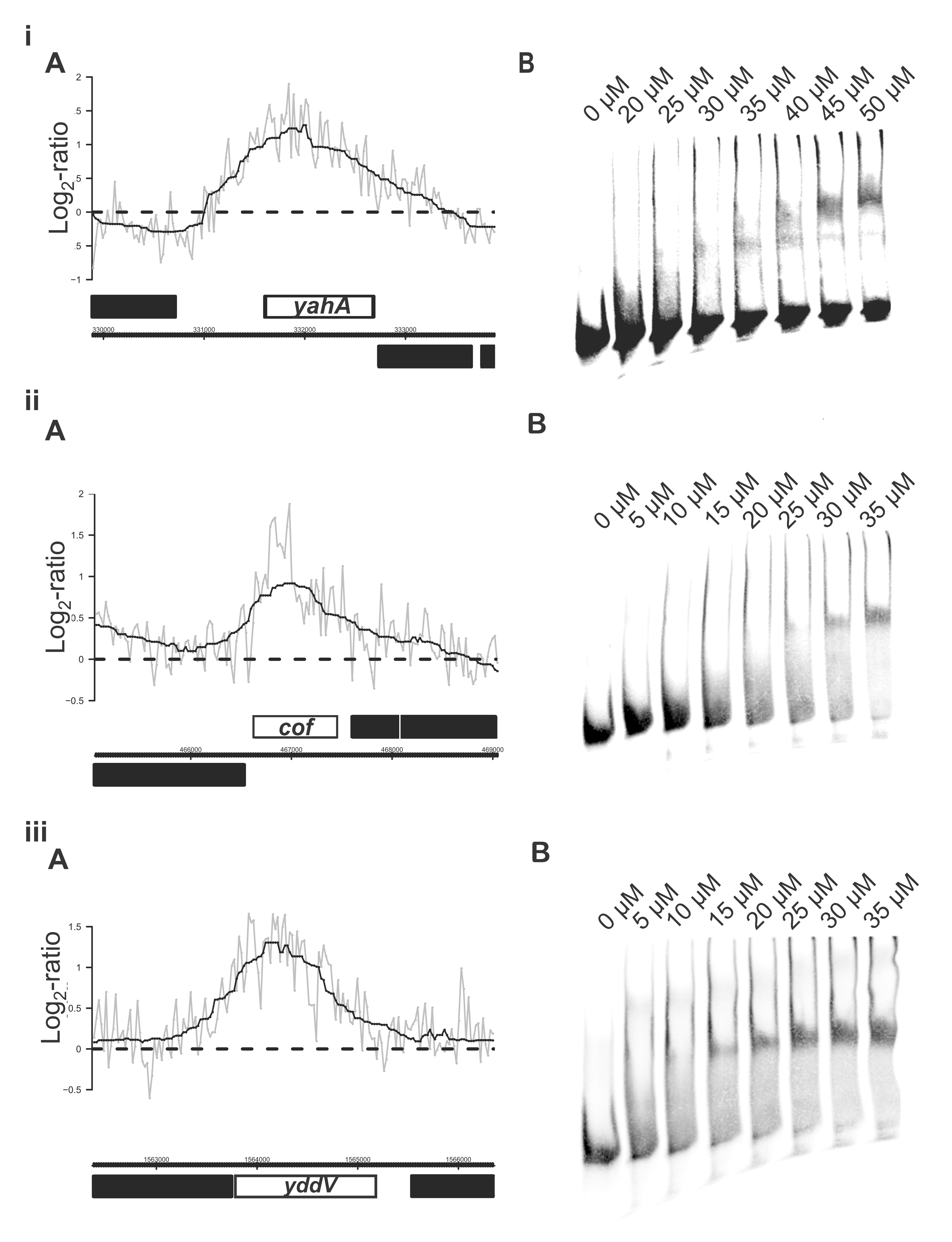

Supplement: Figure S4 — Novel PhoB bindings located in the coding regions may participate in gene regulation. The three genes, yahA (i), cof (ii), and yddV (iii), were shown to be differentially expressed in our gene expression microarray analysis. From our ChIP-chip analysis, significant PhoB binding peaks were detected and the putative PhoB binding motifs were also identified within the coding regions (panel A). For further investigation of PhoB bindings, the in vitro binding assays were carried out by gel mobility shift assay (panel B, see Methods for details). The results demonstrate that PhoB binds to the coding regions of the three genes despite the weak binding to yahA. (TIF) [file pone.0047314.s004.tif]

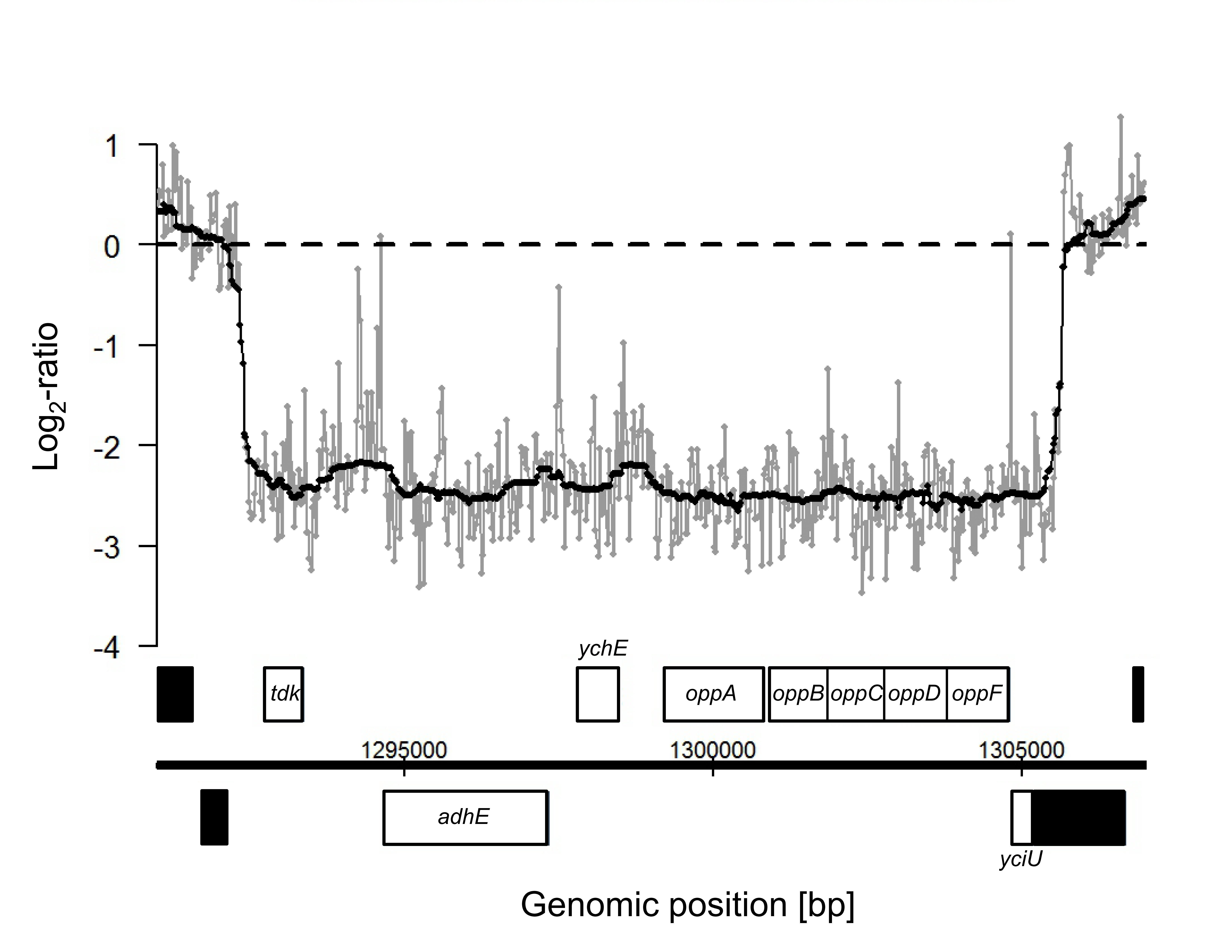

Supplement: Figure S5 — A non-preferred binding region of PhoB. In addition to PhoB binding regions, there is a long region in which the PhoB binding signals are lower than background noises. This region ranges from the genomic location of 1292000 to 1306000 bps. The y-axis represents the log2 ratio (grey line) and the smoothed ratio (black line) of the normalized Cy5 signal (MG1655_PhoB_FLAG) divided by the normalized Cy3 signal (MG1655) after averaging our triplicate results. In order to show the boundary of this non-preferred binding region, the genomic region from 1291000 to 1207000 bps is plotted. Genes located in this region are shown at the bottom of the plot. The pseudogene, insZ, located between tdk and adhE is not shown. All genes, tdk, insZ, adhE, ychE, oppABCDF and yciU were up-regulated with PhoB activity. (TIF) [file pone.0047314.s005.tif]
