# Supplementary material for: Genome-Wide PhoB Binding and Gene Expression Profiles Reveal the Hierarchical Gene Regulatory Network of Phosphate Starvation in Escherichia coli
Source: PLoS One. 2012 Oct 5;7(10):e47314. doi: 10.1371/journal.pone.0047314 (PMC3465305; doi:10.1371/journal.pone.0047314)
Supplement: Table S1 — List of differentially expressed genes identified from the microarray analysis. (DOCX) [file pone.0047314.s006.docx]

**Table S1.** Differentially expressed genes identified from the microarray analysis.

| Gene name | Log_2_-ratio | Adjusted P-value | COG | Gene product |
| --- | --- | --- | --- | --- |
| *phnD* | 9.696186 | 1.23E-05 | COG3221P | phosphonate/organophosphate ester transporter subunit |
| *adhE* | 9.161698 | 2.68E-05 | COG1454C, COG1012C | fused acetaldehyde-CoA dehydrogenase/iron-dependent alcohol dehydrogenase/pyruvate-formate lyase deactivase |
| *phnE* | 8.595734 | 7.97E-06 | - | pseudo gene |
| *phoB* | 8.517565 | 7.97E-06 | COG0745TK | DNA-binding response regulator in two-component regulatory system with PhoR (or CreC) |
| *phnC* | 8.264802 | 1.23E-05 | COG3638P | phosphonate/organophosphate ester transporter subunit |
| *phoE* | 8.216763 | 7.97E-06 | COG3203M | outer membrane phosphoporin protein E |
| *oppA* | 8.109159 | 1.23E-05 | COG4166E | oligopeptide transporter subunit |
| *phnH* | 7.787983 | 3.36E-05 | COG3625P | carbon-phosphorus lyase complex subunit |
| *phnF* | 7.596289 | 1.23E-05 | COG2188K | predicted DNA-binding transcriptional regulator of phosphonate uptake and biodegradation |
| *phnJ* | 7.384597 | 0.00018 | COG3627P | carbon-phosphorus lyase complex subunit |
| *phnI* | 7.384295 | 0.000114 | COG3626P | carbon-phosphorus lyase complex subunit |
| *oppC* | 7.183556 | 0.000145 | COG1173EP | oligopeptide transporter subunit |
| *oppD* | 7.114934 | 1.23E-05 | COG0444EP | oligopeptide transporter subunit |
| *oppB* | 6.677597 | 0.000193 | COG0601EP | oligopeptide transporter subunit |
| *phnG* | 6.613281 | 1.23E-05 | COG3624P | carbon-phosphorus lyase complex subunit |
| *phnL* | 6.601807 | 4.88E-05 | COG4778P | carbon-phosphorus lyase complex subunit |
| *pstS* | 6.200781 | 1.82E-05 | COG0226P | phosphate transporter subunit |
| *phnM* | 6.153311 | 4.88E-05 | COG3454P | carbon-phosphorus lyase complex subunit |
| *yciU* | 6.119418 | 0.000236 | COG3099S | predicted protein |
| *phoA* | 6.095461 | 1.72E-05 | COG1785P | bacterial alkaline phosphatase |
| *pstC* | 6.076072 | 2.76E-05 | COG0573P | phosphate transporter subunit |
| *oppF* | 6.069908 | 9.47E-05 | COG4608E | oligopeptide transporter subunit |
| *phnK* | 5.703785 | 1.82E-05 | COG4107P | carbon-phosphorus lyase complex subunit |
| *yibD* | 5.458011 | 1.82E-05 | COG0463M | predicted glycosyl transferase |
| *pstA* | 5.348056 | 4.88E-05 | COG0581P | phosphate transporter subunit |
| *phnN* | 4.964476 | 3.56E-05 | COG3709P | ribose 1,5-bisphosphokinase |
| *gfcE* | 4.891477 | 0.000236 | COG1596M | predicted exopolysaccharide export protein |
| *yfaH* | 4.522178 | 4.66E-05 | - | pseudo gene |
| *phoU* | 4.136269 | 4.88E-05 | COG0704P | negative regulator of PhoR/PhoB two-component regulator |
| *pstB* | 4.127005 | 5.10E-05 | COG1117P | phosphate transporter subunit |
| *cdh* | 4.042013 | 0.000199 | COG2134I | CDP-diacylglycerol phosphotidylhydrolase |
| *tdk* | 3.602688 | 0.000158 | COG1435F | thymidine kinase/deoxyuridine kinase |
| *cpsG* | 3.568236 | 0.000194 | COG1109G | phosphomannomutase |
| *psiF* | 3.272639 | 0.000269 | - | conserved protein |
| *yebE* | 3.147401 | 0.000145 | COG2979S | conserved protein |
| *yiaD* | 3.106061 | 0.000822 | COG2885M | predicted outer membrane lipoprotien |
| *ulaG* | 3.091282 | 0.000376 | COG2220R | predicted L-ascorbate 6-phosphate lactonase |
| *ytfK* | 3.06661 | 0.000357 | COG1117P | conserved protein |
| *etp* | 3.059823 | 0.000194 | COG0394T | phosphotyrosine-protein phosphatase |
| *adrA* | 3.042272 | 0.000908 | COG2199T | predicted diguanylate cyclase |
| *cusF* | 3.012128 | 0.000927 | COG5569S | periplasmic copper-binding protein |
| *ychE* | 2.998274 | 0.000247 | COG2095U | predicted inner membrane protein |
| *hcaF* | 2.87959 | 0.000247 | COG5517Q | 3-phenylpropionate dioxygenase, small (beta) subunit |
| *hcaE* | 2.813185 | 0.000207 | COG4638PR | 3-phenylpropionate dioxygenase, large (alpha) subunit |
| *wcaK* | 2.779953 | 0.000274 | COG2327S | predicted pyruvyl transferase |
| *cusC* | 2.75251 | 0.001865 | COG1538MU | copper/silver efflux system, outer membrane component |
| *wcaJ* | 2.720479 | 0.01683 | COG2148M | predicted UDP-glucose lipid carrier transferase |
| *ugpA* | 2.556868 | 0.000383 | COG1175G | glycerol-3-phosphate transporter subunit |
| *ygaY* | 2.508015 | 0.001134 | - | pseudo gene |
| *ugpE* | 2.494989 | 0.00117 | COG0395G | glycerol-3-phosphate transporter subunit |
| *gldA* | 2.405301 | 0.000381 | COG0371C | glycerol dehydrogenase, NAD |
| *yfiD* | 2.399613 | 0.00365 | COG3445R | pyruvate formate lyase subunit |
| *hcaB* | 2.398775 | 0.000385 | COG1028IQR | 2,3-dihydroxy-2,3-dihydrophenylpropionate dehydrogenase |
| *ynfK* | 2.339678 | 0.034508 | COG0132H | predicted dethiobiotin synthetase |
| *hcaC* | 2.31408 | 0.000496 | COG2146PR | 3-phenylpropionate dioxygenase, predicted ferredoxin subunit |
| *yhbU* | 2.247405 | 0.021933 | COG0826O | predicted peptidase (collagenase-like) |
| *ugpC* | 2.225338 | 0.000642 | COG3839G | glycerol-3-phosphate transporter subunit |
| *wcaL* | 2.194687 | 0.000677 | COG0438M | predicted glycosyl transferase |
| *wzxC* | 2.181164 | 0.027908 | COG2244R | colanic acid exporter |
| *hcaD* | 2.17568 | 0.00206 | COG0446R | phenylpropionate dioxygenase, ferredoxin reductase subunit |
| *ahpF* | 2.172446 | 0.000747 | COG3634O | alkyl hydroperoxide reductase, F52a subunit, FAD/NAD(P)-binding |
| *bglG* | 2.150663 | 0.002671 | COG3711K | transcriptional antiterminator of the bgl operon |
| *psiE* | 2.148127 | 0.000642 | COG3223S | predicted phosphate starvation inducible protein |
| *mipA* | 2.086954 | 0.000704 | COG3713M | scaffolding protein for murein synthesizing machinery |
| *insZ* | 1.960157 | 0.007524 | - | pseudo gene |
| *iraP* | 1.939974 | 0.004276 | - | predicted protein |
| *etk* | 1.901619 | 0.006093 | COG3206M, COG0489D | cryptic autophosphorylating protein tyrosine kinase Etk |
| *ybfA* | 1.892429 | 0.010965 | - | predicted protein |
| *phoR* | 1.854057 | 0.006183 | COG0642T | sensory histidine kinase in two-component regulatory system with PhoB |
| *amn* | 1.851143 | 0.002585 | COG0775F | AMP nucleosidase |
| *rimL* | 1.845323 | 0.01361 | COG1670J | ribosomal-protein-L7/L12-serine acetyltransferase |
| *ansP* | 1.821144 | 0.001134 | COG1113E | L-asparagine transporter |
| *cnu* | 1.76844 | 0.005143 | - | predicted regulator |
| *phnP* | 1.744313 | 0.001347 | COG1235R | carbon-phosphorus lyase complex accessory protein |
| *yoeA* | 1.712194 | 0.018489 | - | pseudo gene |
| *pepE* | 1.705555 | 0.002939 | COG3340E | (alpha)-aspartyl dipeptidase |
| *cusB* | 1.701431 | 0.002699 | COG0845M | copper/silver efflux system, membrane fusion protein |
| *gntK* | 1.682546 | 0.006183 | COG3265G | gluconate kinase 2 |
| *phnO* | 1.646708 | 0.002001 | COG0454KR | predicted acyltransferase with acyl-CoA N-acyltransferase domain |
| *ybhJ* | 1.624775 | 0.005143 | COG1048C | predicted hydratase |
| *yoaI* | 1.578128 | 0.017985 | - | predicted protein |
| *yedX* | 1.561523 | 0.00654 | COG2351R | conserved protein |
| *yeeN* | 1.556345 | 0.005698 | COG0217S | conserved protein |
| *agaD* | 1.54375 | 0.01683 | COG3716G | N-acetylgalactosamine-specific enzyme IID component of PTS |
| *ugd* | 1.518059 | 0.004009 | COG1004M | UDP-glucose 6-dehydrogenase |
| *ydjY* | 1.480503 | 0.004175 | - | predicted protein |
| *rpsF* | 1.432454 | 0.006518 | - | 30S ribosomal subunit protein S6 |
| *yeeV* | 1.409969 | 0.0381 | - | CP4-44 prophage; toxin of the YeeV-YeeU toxin-antitoxin system |
| *ugpQ* | 1.373525 | 0.009648 | COG0584C | glycerophosphodiester phosphodiesterase, cytosolic |
| *rihB* | 1.323996 | 0.018256 | COG1957F | ribonucleoside hydrolase 2 |
| *pagP* | 1.322204 | 0.017066 | - | palmitoyl transferase for Lipid A |
| *lysA* | 1.321294 | 0.008927 | COG0019E | diaminopimelate decarboxylase, PLP-binding |
| *cybC* | 1.317565 | 0.016195 | - | pseudo gene |
| *gcvB* | 1.316863 | 0.005143 | - | ncRNA |
| *pphA* | 1.313556 | 0.009594 | COG0639T | serine/threonine-specific protein phosphatase 1 |
| *mdtD* | 1.309752 | 0.036067 | COG0477GEPR | multidrug efflux system protein |
| *yeaE* | 1.308537 | 0.00779 | COG0656R | predicted oxidoreductase |
| *ugpB* | 1.304235 | 0.00654 | COG1653G | glycerol-3-phosphate transporter subunit |
| *ompW* | 1.300736 | 0.048366 | COG3047M | outer membrane protein W |
| *cbrB* | 1.293629 | 0.034259 | - | predicted inner membrane protein |
| *setB* | 1.293034 | 0.016616 | COG0477GEPR | lactose/glucose efflux system |
| *wcaM* | 1.289338 | 0.005184 | - | predicted colanic acid biosynthesis protein |
| *yzgL* | 1.27516 | 0.010903 | - | pseudo gene |
| *fliQ* | 1.274616 | 0.02032 | COG1987NU | flagellar biosynthesis protein |
| *mdtB* | 1.228773 | 0.006226 | COG0841V | multidrug efflux system, subunit B |
| *nohB* | 1.226569 | 0.045217 | COG4220L | DLP12 prophage; DNA packaging protein |
| *tsx* | 1.175399 | 0.010832 | COG3248M | nucleoside channel, receptor of phage T6 and colicin K |
| *paaC* | 1.16549 | 0.017985 | COG3396S | predicted multicomponent oxygenase/reductase subunit for phenylacetic acid degradation |
| *cbrA* | 1.161419 | 0.02032 | COG0644C | predicted oxidoreductase with FAD/NAD(P)-binding domain |
| *ybaL* | 1.159097 | 0.014161 | COG1226P, COG4651P | predicted transporter with NAD(P)-binding Rossmann-fold domain |
| *frdA* | 1.151785 | 0.020714 | COG1053C | fumarate reductase (anaerobic) catalytic and NAD/flavoprotein subunit |
| *ydfH* | 1.150524 | 0.009189 | COG1802K | predicted DNA-binding transcriptional regulator |
| *yqeF* | 1.143797 | 0.019221 | COG0183I | predicted acyltransferase |
| *yhbV* | 1.136968 | 0.018666 | COG0826O | predicted protease |
| *cusS* | 1.117893 | 0.020731 | COG0642T | sensory histidine kinase in two-component regulatory system with CusR, senses copper ions |
| *rpoH* | 1.103284 | 0.014544 | COG0568K | RNA polymerase, sigma 32 (sigma H) factor |
| *ydaG* | 1.086369 | 0.021933 | - | Rac prophage; predicted protein |
| *ynjE* | 1.077066 | 0.038933 | COG2897P | predicted thiosulfate sulfur transferase |
| *ytfJ* | 1.073297 | 0.028906 | COG3054R | predicted transcriptional regulator |
| *cmr* | 1.070704 | 0.016571 | COG0477GEPR | multidrug efflux system protein |
| *paaK* | 1.059308 | 0.041382 | COG1541H | phenylacetyl-CoA ligase |
| *ychM* | 1.050056 | 0.031457 | COG0659P | predicted transporter |
| *eamB* | 1.039986 | 0.040527 | COG1280E | neutral amino-acid efflux system |
| *cusR* | 1.030724 | 0.041815 | COG0745TK | DNA-binding response regulator in two-component regulatory system with CusS |
| *yjeI* | 1.008567 | 0.013638 | - | conserved protein |
| *paaD* | 1.006984 | 0.031573 | COG2151R | predicted multicomponent oxygenase/reductase subunit for phenylacetic acid degradation |
| *ynfE* | 1.005918 | 0.014634 | COG0243C | oxidoreductase subunit |
| *yjiY* | 0.999981 | 0.012224 | COG1966T | predicted inner membrane protein |
| *cof* | 0.998341 | 0.028451 | COG0561R | thiamin pyrimidine pyrophosphate hydrolase |
| *agaC* | 0.990256 | 0.043315 | COG3715G | N-acetylgalactosamine-specific enzyme IIC component of PTS |
| *ydhC* | 0.981435 | 0.020703 | COG0477GEPR | predicted transporter |
| *yojO* | 0.979706 | 0.020731 | COG1626G | hypothetical protein |
| *mdtA* | 0.972419 | 0.04965 | COG0845M | multidrug efflux system, subunit A |
| *dos* | 0.9704 | 0.020495 | COG2200T, COG2202T, COG2199T | cAMP phosphodiesterase, heme-regulated |
| *mltD* | 0.949192 | 0.028906 | COG0741M | predicted membrane-bound lytic murein transglycosylase D |
| *cusA* | 0.946604 | 0.017131 | COG3696P | copper/silver efflux system, membrane component |
| *tonB* | 0.944233 | 0.021171 | COG0810M | membrane spanning protein in TonB-ExbB-ExbD complex |
| *ahpC* | 0.941986 | 0.017351 | COG0450O | alkyl hydroperoxide reductase, C22 subunit |
| *ydiI* | 0.933231 | 0.016539 | COG2050Q | conserved protein |
| *ychQ* | 0.894706 | 0.038403 | COG3094S | predicted transcriptional regulator |
| *rep* | 0.893077 | 0.019433 | COG0210L | DNA helicase and single-stranded DNA-dependent ATPase |
| *ycfS* | 0.884816 | 0.034508 | COG1376S | L,D-transpeptidase linking Lpp to murein |
| *cbrC* | 0.877454 | 0.025934 | COG3196S | conserved protein |
| *yjiM* | 0.858404 | 0.035667 | COG1775E | predicted 2-hydroxyglutaryl-CoA dehydratase |
| *bssR* | 0.853418 | 0.0416 | - | conserved protein |
| *norV* | 0.845274 | 0.030086 | COG1773C, COG0426C | flavorubredoxin oxidoreductase |
| *argP* | 0.842884 | 0.032112 | COG0583K | DNA-binding transcriptional activator, replication initiation inhibitor |
| *yjeT* | 0.840877 | 0.027908 | COG3242S | conserved inner membrane protein |
| *ulaB* | 0.83243 | 0.046573 | COG3414G | L-ascorbate-specific enzyme IIB component of PTS |
| *nrdG* | 0.814828 | 0.038801 | COG0602O | anaerobic ribonucleotide reductase activating protein |
| *yqgB* | 0.813445 | 0.034259 | - | predicted protein |
| *essD* | 0.803324 | 0.03627 | - | DLP12 prophage; predicted phage lysis protein |
| *argF* | 0.792025 | 0.031101 | COG0078E | CP4-6 prophage; ornithine carbamoyltransferase 2, chain F |
| *yfiF* | 0.787655 | 0.029966 | COG0566J | predicted methyltransferase |
| *acpH* | 0.785329 | 0.046142 | COG3124S | conserved protein |
| *yahA* | 0.781445 | 0.038797 | COG2200T, COG2197TK | predicted DNA-binding transcriptional regulator |
| *gltP* | 0.779155 | 0.037845 | COG1301C | glutamate/aspartate:proton symporter |
| *usg* | 0.777226 | 0.039905 | COG0136E | predicted semialdehyde dehydrogenase |
| *yqjF* | 0.770876 | 0.034259 | COG2259S | predicted quinol oxidase subunit |
| *mdtC* | 0.756582 | 0.029966 | COG0841V | multidrug efflux system, subunit C |
| *puuR* | 0.755099 | 0.035602 | COG1396K | DNA-binding transcriptional repressor |
| *brnQ* | 0.754006 | 0.039474 | COG1114E | predicted branched chain amino acid transporter (LIV-II) |
| *appC* | 0.735445 | 0.044445 | COG1271C | cytochrome bd-II oxidase, subunit I |
| *nepI* | 0.733723 | 0.036963 | COG2814G | predicted transporter |
| *yncK* | 0.728575 | 0.048306 | - | pseudo gene |
| *ydiJ* | 0.726989 | 0.042174 | COG0277C, COG0247C | predicted FAD-linked oxidoreductase |
| *ynfF* | 0.726874 | 0.040974 | - | oxidoreductase subunit |
| *nagB* | 0.71296 | 0.0416 | COG0363G | glucosamine-6-phosphate deaminase |
| *yggW* | 0.703945 | 0.049094 | COG0635H | predicted oxidoreductase |
| *yddV* | 0.687892 | 0.046142 | COG2199T | predicted diguanylate cyclase |
| *uxaB* | 0.681254 | 0.040272 | COG0246G | altronate oxidoreductase, NAD-dependent |
| *ydgK* | 0.679441 | 0.049466 | - | conserved inner membrane protein |
| *nagA* | 0.676797 | 0.04965 | COG1820G | N-acetylglucosamine-6-phosphate deacetylase |
| *micF* | 0.658004 | 0.043789 | - | ncRNA |
| *ybgH* | 0.657072 | 0.046106 | COG3104E | predicted transporter |
| *glgA* | 0.651134 | 0.049146 | COG0297G | glycogen synthase |
| *rlmB* | 0.638655 | 0.049618 | COG0566J | 23S rRNA (Gm2251)-methyltransferase |
| *sbcD* | -0.64318 | 0.04857 | COG0420L | exonuclease, dsDNA, ATP-dependent |
| *icd* | -0.64651 | 0.048306 | COG0538C | e14 prophage; isocitrate dehydrogenase, specific for NADP+ |
| *ycjN* | -0.65379 | 0.045136 | COG1653G | predicted sugar transporter subunit: periplasmic-binding component of ABC superfamily |
| *mrr* | -0.66047 | 0.046142 | COG1715V | methylated adenine and cytosine restriction protein |
| *yoaB* | -0.67622 | 0.045442 | COG0251J | conserved protein |
| *sbcC* | -0.67697 | 0.044332 | COG0419L | exonuclease, dsDNA, ATP-dependent |
| *mnmC* | -0.68622 | 0.040974 | COG0665E, COG4121S | fused 5-methylaminomethyl-2-thiouridine-forming enzyme methyltransferase/FAD-dependent demodification enzyme |
| *ybeM* | -0.70399 | 0.044332 | - | pseudo gene |
| *mdaB* | -0.70479 | 0.039452 | COG2249R | NADPH quinone reductase |
| *gatC* | -0.70733 | 0.046625 | COG3775G | galactitol-specific enzyme IIC component of PTS |
| *atpI* | -0.70884 | 0.036921 | - | ATP synthase, membrane-bound accesory subunit |
| *yidB* | -0.71031 | 0.038403 | COG3753S | conserved protein |
| *ompF* | -0.71035 | 0.039452 | COG3203M | outer membrane porin 1a (Ia;b;F) |
| *malP* | -0.71256 | 0.042174 | COG0058G | maltodextrin phosphorylase |
| *yffQ* | -0.72245 | 0.046983 | - | CPZ-55 prophage; predicted protein |
| *entC* | -0.73484 | 0.035438 | COG1169HQ | isochorismate synthase 1 |
| *dgt* | -0.73576 | 0.038403 | COG0232F | deoxyguanosine triphosphate triphosphohydrolase |
| *ycaK* | -0.73769 | 0.0381 | COG2249R | conserved protein |
| *betT* | -0.73861 | 0.045442 | COG1292M | choline transporter of high affinity |
| *yfgH* | -0.73877 | 0.046196 | - | predicted outer membrane lipoprotein |
| *ygcH* | -0.74729 | 0.036921 | - | predicted protein |
| *ykgJ* | -0.7521 | 0.039706 | COG0727R | predicted ferredoxin |
| *rbsA* | -0.75483 | 0.04857 | COG1129G | fused D-ribose transporter subunits of ABC superfamily: ATP-binding components |
| *hmp* | -0.75673 | 0.032433 | COG1018C, COG1017C | fused nitric oxide dioxygenase/dihydropteridine reductase 2 |
| *yhhM* | -0.76352 | 0.049771 | - | conserved protein |
| *fadA* | -0.77305 | 0.046196 | COG0183I | 3-ketoacyl-CoA thiolase (thiolase I) |
| *yfcV* | -0.77467 | 0.036001 | - | predicted fimbrial-like adhesin protein |
| *ycjO* | -0.78123 | 0.04857 | COG1175G | predicted sugar transporter subunit: membrane component of ABC superfamily |
| *yhjC* | -0.78268 | 0.028802 | COG0583K | predicted DNA-binding transcriptional regulator |
| *yeiQ* | -0.78435 | 0.031457 | COG0246G | predicted dehydrogenase, NAD-dependent |
| *pspA* | -0.78693 | 0.029966 | COG1842KT | regulatory protein for phage-shock-protein operon |
| *gatB* | -0.78919 | 0.028013 | COG3414G | galactitol-specific enzyme IIB component of PTS |
| *betI* | -0.78939 | 0.027908 | COG1309K | DNA-binding transcriptional repressor |
| *cspD* | -0.79499 | 0.046983 | COG1278K | cold shock protein homolog |
| *gpt* | -0.80103 | 0.044332 | COG0503F | guanine-hypoxanthine phosphoribosyltransferase |
| *iscR* | -0.80618 | 0.049618 | COG1959K | DNA-binding transcriptional repressor |
| *intS* | -0.80699 | 0.02851 | COG0582L | CPS-53 (KpLE1) prophage; predicted prophage CPS-53 integrase |
| *rbbA* | -0.82746 | 0.037978 | COG1131V, COG0842V | fused ribosome-associated ATPase: ATP-binding protein/ATP-binding protein/predicted membrane protein |
| *tpr* | -0.82818 | 0.049771 | - | predicted protamine-like protein |
| *rfaD* | -0.83047 | 0.04003 | COG0451MG | ADP-L-glycero-D-mannoheptose-6-epimerase, NAD(P)-binding |
| *panB* | -0.83181 | 0.02508 | COG0413H | 3-methyl-2-oxobutanoate hydroxymethyltransferase |
| *uxuA* | -0.83541 | 0.031807 | COG1312G | mannonate hydrolase |
| *argT* | -0.84221 | 0.037845 | COG0834ET | lysine/arginine/ornithine transporter subunit |
| *pyrB* | -0.84381 | 0.021157 | COG0540F | aspartate carbamoyltransferase, catalytic subunit |
| *glgS* | -0.84965 | 0.037845 | - | predicted glycogen synthesis protein |
| *rmf* | -0.85486 | 0.021955 | COG3130J | ribosome modulation factor |
| *ytjA* | -0.85693 | 0.02122 | - | predicted protein |
| *yjcH* | -0.86477 | 0.02032 | COG3162S | conserved inner membrane protein involved in acetate transport |
| *uspF* | -0.86548 | 0.035438 | COG0589T | stress-induced protein, ATP-binding protein |
| *ybiU* | -0.86927 | 0.021171 | - | predicted protein |
| *hisJ* | -0.88115 | 0.022401 | COG0834ET | histidine/lysine/arginine/ornithine transporter subunit |
| *astD* | -0.88413 | 0.018341 | COG1012C | succinylglutamic semialdehyde dehydrogenase |
| *yicE* | -0.88948 | 0.041048 | COG2233F | predicted transporter |
| *puuC* | -0.89102 | 0.022446 | COG1012C | gamma-Glu-gamma-aminobutyraldehyde dehydrogenase, NAD(P)H-dependent |
| *yfhH* | -0.893 | 0.042174 | COG1737K | predicted DNA-binding transcriptional regulator |
| *puuB* | -0.89521 | 0.024556 | COG0665E | gamma-Glu-putrescine oxidase, FAD/NAD(P)-binding |
| *fabB* | -0.90929 | 0.025414 | COG0304IQ | 3-oxoacyl-[acyl-carrier-protein] synthase I |
| *pspB* | -0.91202 | 0.049022 | - | DNA-binding transcriptional regulator of psp operon |
| *fadB* | -0.92349 | 0.024517 | COG1024I, COG1250I | fused 3-hydroxybutyryl-CoA epimerase/delta(3)-cis-delta(2)-trans-enoyl-CoA isomerase/enoyl-CoA hydratase/3-hydroxyacyl-CoA dehydrogenase |
| *yegH* | -0.92375 | 0.016539 | COG1253R, COG0861P | fused predicted membrane protein/predicted membrane protein |
| *fbp* | -0.93359 | 0.016182 | COG0158G | fructose-1,6-bisphosphatase I |
| *puuE* | -0.93575 | 0.037845 | COG0160E | GABA aminotransferase, PLP-dependent |
| *yagN* | -0.94837 | 0.023241 | - | CP4-6 prophage; predicted protein |
| *kgtP* | -0.95095 | 0.04003 | COG0477GEPR | alpha-ketoglutarate transporter |
| *dcuS* | -0.95661 | 0.018341 | COG3290T | sensory histidine kinase in two-component regulatory system with DcuR, regulator of anaerobic fumarate respiration |
| *malG* | -0.97602 | 0.035846 | COG3833G | maltose transporter subunit |
| *astC* | -1.0032 | 0.024942 | COG4992E | succinylornithine transaminase, PLP-dependent |
| *fimA* | -1.01413 | 0.039762 | COG3539NU | major type 1 subunit fimbrin (pilin) |
| *yjgF* | -1.02678 | 0.012122 | COG0251J | ketoacid-binding protein |
| *waaA* | -1.03206 | 0.017459 | COG1519M | 3-deoxy-D-manno-octulosonic-acid transferase (KDO transferase) |
| *yhiI* | -1.03421 | 0.049771 | COG0845M | predicted HlyD family secretion protein |
| *mgtA* | -1.05343 | 0.015611 | COG0474P | magnesium transporter |
| *rsd* | -1.05904 | 0.033703 | COG3160K | stationary phase protein, binds sigma 70 RNA polymerase subunit |
| *ryeA* | -1.06297 | 0.017985 | - | ncRNA |
| *araC* | -1.07011 | 0.016863 | COG2207K | DNA-binding transcriptional dual regulator |
| *crl* | -1.10072 | 0.015579 | - | DNA-binding transcriptional regulator |
| *acs* | -1.102 | 0.012122 | COG0365I | acetyl-CoA synthetase |
| *rbsD* | -1.1438 | 0.045442 | COG1869G | predicted cytoplasmic sugar-binding protein |
| *sdhC* | -1.15058 | 0.049214 | COG2009C | succinate dehydrogenase, membrane subunit, binds cytochrome b556 |
| *astA* | -1.15815 | 0.00779 | COG3138E | arginine succinyltransferase |
| *isrB* | -1.15825 | 0.025934 | - | ncRNA |
| *proC* | -1.16328 | 0.008927 | COG0345E | pyrroline-5-carboxylate reductase, NAD(P)-binding |
| *glcC* | -1.17655 | 0.024517 | COG2186K | DNA-binding transcriptional dual regulator, glycolate-binding |
| *fadL* | -1.18783 | 0.009227 | COG2067I | long-chain fatty acid outer membrane transporter |
| *aldA* | -1.27723 | 0.021933 | COG1012C | aldehyde dehydrogenase A, NAD-linked |
| *glcB* | -1.28385 | 0.009375 | COG2225C | malate synthase G |
| *malM* | -1.29116 | 0.009172 | - | maltose regulon periplasmic protein |
| *glcG* | -1.31489 | 0.004478 | COG3193R | conserved protein |
| *malF* | -1.37861 | 0.003961 | COG1175G | maltose transporter subunit |
| *osmY* | -1.38531 | 0.008828 | COG2823R | periplasmic protein |
| *bdm* | -1.39479 | 0.014102 | - | biofilm-dependent modulation protein |
| *yqfA* | -1.42649 | 0.010832 | COG1272R | predicted oxidoreductase, inner membrane subunit |
| *torY* | -1.44604 | 0.028636 | COG3005C | TMAO reductase III (TorYZ), cytochrome c-type subunit |
| *feaR* | -1.47662 | 0.031573 | COG2207K | DNA-binding transcriptional dual regulator |
| *prpR* | -1.48109 | 0.01213 | COG1221KT, COG3829KT | DNA-binding transcriptional activator |
| *glcA* | -1.56927 | 0.010366 | COG1620C | glycolate transporter |
| *glcF* | -1.58289 | 0.02285 | COG0247C | glycolate oxidase iron-sulfur subunit |
| *nmpC* | -1.59871 | 0.047299 | - | pseudo gene |
| *uspG* | -1.64425 | 0.008612 | COG0589T | universal stress protein UP12 |
| *glcD* | -1.65798 | 0.002585 | COG0277C | glycolate oxidase subunit, FAD-linked |
