# Supplementary material for: Genome-Wide PhoB Binding and Gene Expression Profiles Reveal the Hierarchical Gene Regulatory Network of Phosphate Starvation in Escherichia coli
Source: PLoS One. 2012 Oct 5;7(10):e47314. doi: 10.1371/journal.pone.0047314 (PMC3465305; doi:10.1371/journal.pone.0047314)
Supplement: Table S2 — List of plasmids used in this study. (DOC) [file pone.0047314.s007.doc]

**Table S2.** Plasmids used in this study.

| **Name** | **Description^*^** | **Reference or source** |
| --- | --- | --- |
| pKD46 | Rec recombinase expression vector, Rep^ts^, Ap^r^ | [1] |
|  |  |  |
|  |  |  |
|  |  |  |
| pSUB11 | Plasmid with a 24 nt FLAG sequence and FRT sites flanking a kanamycin cassette | [2] |
| pCP20 | FLP^+^, λ cI857^+^, λ P_R_ Rep^ts^, Ap^r^, Cm^r^ | [2] |
| pET21d(+) | Vectors with T7 promoter | Novagen |
| pET21d(+)*phoB-his_(6x)_* | pET21d(+) derivative, inducing expression of PhoB-His_(6x)_ by IPTG | This study |
| pGL3 | promoterless reporter vector, with a *luc* reporter gene, f1 *ori*, Ap^r^ | Promega |
| pGL3_phoB | pGL3 derivative, containing an upstream region of *phoB* | This study |
| pGL3_feaR | pGL3 derivative, containing an upstream region of *feaR* | This study |
| pGL3_prpR | pGL3 derivative, containing an upstream region of *prpR* | This study |
| pGL3_ydfH | pGL3 derivative, containing an upstream region of *ydfH* | This study |
| pGL3_yedX | pGL3 derivative, containing an upstream region of *yedX* | This study |
| pGL3_cusR | pGL3 derivative, containing an upstream region of *cusR* | This study |
| pGL3_mipA | pGL3 derivative, containing an upstream region of *mipA* | This study |
| pGL3_cusC | pGL3 derivative, containing an upstream region of *cusC* | This study |
| pGL3_yhjC | pGL3 derivative, containing an upstream region of *yhjC* | This study |
| pGL3_yegH | pGL3 derivative, containing an upstream region of *yegH* | This study |
| pGL3_sbcD | pGL3 vector derivative, containing an upstream region of *sbcD* | This study |

Abbreviations: Km^r^, kanamycin resistance; Ap^r^, ampicillin resistance; Cm^r^, chloramphenicol resistance.

^*^For the construction of pGL3 derivative plasmids, the upstream regions used were around 500 bps in front of the translation start sites of each structural gene. The complete list of primers used for the construction is shown in supplementary Table 2.

**References**

1. Datsenko KA, Wanner BL: **One-step inactivation of chromosomal genes in Escherichia coli K-12 using PCR products.** *Proc Natl Acad Sci U S A* 2000, **97:**6640-6645.

2. Uzzau S, Figueroa-Bossi N, Rubino S, Bossi L: **Epitope tagging of chromosomal genes in Salmonella.** *Proc Natl Acad Sci U S A* 2001, **98:**15264-15269.
