# Supplementary material for: Genome-Wide PhoB Binding and Gene Expression Profiles Reveal the Hierarchical Gene Regulatory Network of Phosphate Starvation in Escherichia coli
Source: PLoS One. 2012 Oct 5;7(10):e47314. doi: 10.1371/journal.pone.0047314 (PMC3465305; doi:10.1371/journal.pone.0047314)
Supplement: Table S3 — List of oligonucleotides used in this study. (DOC) [file pone.0047314.s008.doc]

**Table S3.** Oligonucleotides used in this study.

| PCR target | Forward and reverse primer | | | Sequence (5’-3’) |
| --- | --- | --- | --- | --- |
| (a) Primers for PhoB-3xFLAG expressing strain (MG1655_PhoB_FLAG) | | | | |
| *phoB* and pSUB11 | | *phoB*-pSUB11-F | | 5’-GACCGTGCGCGGTACAGGATATCGTTTTTCA ACCCGCTTTGACTACAAAGACCATGACGG-3’ |
|  |  | *phoB*-pSUB11-R | | 5’-ATAAGCCCTGCTCTGCGTCCGATGAGCAAGG CGTTAAAAGCATATGAATATCCTCCTTAG-3’ |
| *phoB* | | *phoB*-check1122-F | | 5’-TGCCAGTCTGAGGTGTGAAG-3’ |
|  |  | *phoB*-check1122-R | | 5’-GGCGCAATAAATTCCAGAAA-3’ |
| (b) Primers for detection of inserts of pGL3 derivatives | | | | |
| pGL3-basic vector | | pGL3_InsChk_F | | 5’-GGTACGGGAGGTACTTGGAGC-3’ |
|  |  | pGL3_InsChK_R | | 5’-GGCCTTTCTTTATGTTTTTGGC-3’ |
| (c) Primers for constructing PhoB-His fusion protein expressing vector | | | | |
| *phoB* | | PhoB_His_F | | 5’-GCCCATGGCGAGACGTATTCTGGTCG-3’ |
|  |  | PhoB_His_R | | 5’-GCAAGCTTAAAGCGGGTTGAAAAACGATATCCTG-3’ |
| (d) Primers for constructing promoter::luciferase fusions^a^ | | | | |
| *phoB* | | phoB_FPB | | 5’-GCGCTAGCACGGTAGTATTGAGGAACGCC-3’ |
|  |  | phoB_RPB | | 5’-GCCCATGGGATTTGCCCTGTTGTAATAAATAGG-3’ |
| *cusC* | | cusC_FPB | | 5’-GCGCTAGCCGCCAGCAGTTCAGCAAA |
|  |  | cusC_RPB | | 5’-GCCCATGGAGGCTCATAATTTCTGGTGATTTTA |
| *cusR* | | cusR_FPB | | 5’-GCGCTAGCTTAAGATTACCGCTCCAGCTGC |
|  |  | cusR_RPB | | 5’-GCCCATGGATTTCCTCCGCATGTTGCC |
| *feaR* | | feaR_FPB | | 5’-GCGCTAGCGTAGCAGAATACGTTCACGCTCTG |
|  |  | feaR_RPB | | 5’-GCCCATGGGGCACGTTTTCGCTCTGT |
| *mipA* | | mipA_FPB | | 5’-GCGCTAGCTGCAGTGTGAACTCTTCGTCCT |
|  |  | mipA_RPB | | 5’-GCCCATGGAATTAATCATTCCTTAAACAAATGTTTAG |
| *prpR* | | prpR_FPB | | 5’-GCGCTAGCTATCCGCATCCACCAGCA |
|  |  | prpR_RPB | | 5’-GCCCATGGAGCGCACCGCAAAGTTAAG |
| *sbcD* | | sbcD_FPB | | 5’-GCGCTAGCTGATATAGTCATCCGCGCCG |
|  |  | sbcD_RPB | | 5’-GCCCATGGAACGGTTCCCTGGCGAAA |
| *ydfH* | | ydfH_FPB | | 5’-GCGCTAGCCGCCGTCTTACCGGGTATG |
|  |  | ydfH_RPB | | 5’-GCCCATGGTCGTTCTTGCTTGTGAGTGAGTTAA |
| *yedX* | | yedX_FPB | | 5’-GCGCTAGCGAATTCAAAGCGTGATGTTGC |
|  |  | yedX_RPB | | 5’-GCCCATGGGTTTATATCCTTGTCATGTGAATGAGT |
| *yegH* | | yegH_FPB | | 5’-GCGCTAGCTGGTCTTTCAGGCATCCAGA |
|  |  | yegH_RPB | | 5’-GCCCATGGAGTAAGTACAAAACCTATTCGTTGTATGA |
| *yhjC* | | yhjC_FPB | | 5’-GCGCTAGCAAAGACTTAAGTAGTGGAAGGGTATTACC |
|  |  | yhjC_RPB | | 5’-GCCCATGGTTCTTTGACCGATTGTTGTTCAC |
| (e) Oligonucleotides^b^ for DNA binding assay | | | | |
| *mipA* | | mipA_p | | 5’-GCGTTACTGGCGAGCCTGGTCTTTACATT  AATTATGCAAAATTTATGGATGAGTTGTTGA-3’ |
|  |  | mipA_c | | 5’-TCAACAACTCATCCATAAATTTTGCATAAT  TAATGTAAAGACCAGGCTCGCCAGTAACGC-3’ |
| *yedX* | | yedX_p | | 5’-ATGTTATAGAAACAGCCTGGTTCATTACAA  AATTGTAATGCTGCTGTAAGGTTACCCTGG-3’ |
|  |  | yedX_c | | 5’-CCAGGGTAACCTTACAGCAGCATTACAAT  TTTGTAATGAACCAGGCTGTTTCTATAACAT-3’ |
| *ydfH* | | ydfH_p | | 5’-GCGTAACTGCCGGGTTATTGCTTGTCACA  AAAAAGTGGTAGACTCATGCAGTTAACTCAC-3’ |
|  |  | ydfH_c | | 5’-GTGAGTTAACTGCATGAGTCTACCACTTT  TTTGTGACAAGCAATAACCCGGCAGTTACGC-3’ |
| *cusR* | | cusR_p | | 5’-TGGCAATCGCTTATTGGCAAAATGACAAT  TTTGTCATTTTTCTGTCACCGGAAAATCAGA-3’ |
|  |  | cusR_c | | 5’-TCTGATTTTCCGGTGACAGAAAAATGACA  AAATTGTCATTTTGCCAATAAGCGATTGCCA-3’ |
| *yhjC* | | yhjC_p | | 5’-TGAGAAATCGCCACATTCGGCATGACAAC  ATTGTGAAACCCGGCATTAGATGTTAGAAAA-3’ |
|  |  | yhjC_c | | 5’-TTTTCTAACATCTAATGCCGGGTTTCACAA  TGTTGTCATGCCGAATGTGGCGATTTCTCA-3’ |
| *yegH* | | yegH_p | | 5’-CGATTTATCCATGATCGAATTGTGACATTT  GTCATACAACGAATAGGTTTTGTACTTACT-3’ |
|  |  | yegH_c | | 5’-AGTAAGTACAAAACCTATTCGTTGTATGAC  AAATGTCACAATTCGATCATGGATAAATCG-3’ |
| *feaR* | | feaR_p | | 5’-TCTGTGAAATGTATTTTTATTGTTGCATTT  GTGTTGCAATAAACGAAGCTAATGAGCCTG-3’ |
|  |  | feaR_c | | 5’-CAGGCTCATTAGCTTCGTTTATTGCAACA  CAAATGCAACAATAAAAATACATTTCACAGA-3’ |
| *prpR* | | prpR_p | | 5’-CCGAATATTGGGTTTAGTCTTGTTTCATAA  TTGTTGCAATGAAACGCGGTGAAACATTGC-3’ |
|  |  | prpR_c | | 5’-GCAATGTTTCACCGCGTTTCATTGCAACA  ATTATGAAACAAGACTAAACCCAATATTCGG-3’ |
| *yahA* | | yahA_p | | 5’-TATCCGCCACGGGGAGTAATAGTCACAGA  TATATTAATGATAATCACTATCACCATATCG-3’ |
|  |  | yahA_c | | 5’-CGATATGGTGATAGTGATTATCATTAATATA  TCTGTGACTATTACTCCCCGTGGCGGATA-3’ |
| *cof* | | cof_p | | 5’-GTTTTCGTTATCAGATAATCGATGTCAAAA  AAATGCCACTCGGCAGCGTCACCAAGATCT-3’ |
|  |  | cof_c | | 5’-AGATCTTGGTGACGCTGCCGAGTGGCATT  TTTTTGACATCGATTATCTGATAACGAAAAC-3’ |
| *yddV* | | yddV_p | | 5’-TTGCGATACGGTATTTCTTATCTGTAATAAA  AATTTCACCCGCAGACTTCTGTTATTCAA-3’ |
|  |  | yddV_c | | 5’-TTGAATAACAGAAGTCTGCGGGTGAAATT  TTTATTACAGATAAGAAATACCGTATCGCAA-3’ |
|  | |  |  | |

^a^For the construction of pGL3 derivative plasmids, the upstream region was around 500 bps in front of the translation start site of each gene.

^b^For each binding candidate, complimentary oligonucleotide of 60 base pairs centered at the corresponding putative binding site were first end-labeled with biotin separately and then annealed before use.
